# Safety profile and surgical outcomes of the endoscopic transorbital approach as a skull base surgical corridor: a systematic review and meta-analysis

**DOI:** 10.1007/s10143-026-04321-x

**Published:** 2026-05-13

**Authors:** Rudolfh Batista Arend, Bruno Zilli Peroni, Filipe Virgilio Ribeiro, Marcos Henrique da Silva Mezzari, Natan Lucca Lima, Daniel Marchi Kieling, Victor Luiz Ferreira Kauer, Henrique Padilha Gnoatto, Daniel Felipe Savaris, Gabriel Cambruzzi, Anderson Silva Corin, Raphael Bertani, Alex Roman, Martin Batista Coutinho da Silva, Antonio Delacy Martini Vial, Pierre-Olivier Champagne, Guilherme Gago

**Affiliations:** 1 School of Medicine, Federal University of Fronteira Sul, Passo Fundo, RS Brazil; 2 School of Medicine, Barão de Mauá Faculty of Medicine, Ribeirão Preto, SP Brazil; 3 School of Medicine, Federal University of Santa Catarina, Araranguá, SC Brazil; 4 School of Medicine, Federal University of Pelotas, Pelotas, RS Brazil; 5 Department of Neurosurgery, Hospital Israelita Albert Einstein, São Paulo, SP Brazil; 6 Department of Neurosurgery, Institute of Neurosurgery and Spine Surgery (INCC), Passo Fundo, RS Brazil; 7 School of Medicine, University of Passo Fundo, Passo Fundo, RS Brazil; 8 Department of Neurosurgery, Irmandade Santa Casa de Misericórdia de Porto Alegre, Hospital São José, Porto Alegre, RS Brazil; 9 Department of Neurosurgery, Université Laval, Laval, QC Canada; 10 Department of Postgraduate Program in Medicine: Surgical Sciences, Universidade Federal do Rio Grande do Sul, Porto Alegre, Rio Grande do Sul Brazil

**Keywords:** ETOA, Meningioma, Neurosurgery, Skull base

## Abstract

Endoscopic techniques have enabled minimally invasive approaches in neurosurgery, providing shorter recovery times and favorable outcomes. Among these, the endoscopic transorbital approach (ETOA) has emerged as a versatile surgical modality. Despite the growing body of evidence, complication rates associated with ETOA have not yet been systematically evaluated. We systematically searched PubMed, Embase, Scopus, and Web of Science up to March 2026. We included studies enrolling ≥ 5 patients who underwent ETOA as the sole surgical modality to treat both skull base and orbital lesions, providing data on early or long-term complications. A single-group meta-analysis was performed using a random-effects model with 95% confidence intervals. Heterogeneity was assessed with the I² statistic and further explored through Baujat plots and sensitivity analyses. A total of 11 observational studies, comprising 269 patients, were included. Overall, 21 different pathologies were reported. Meningioma represented the most frequent lesion (60.6% of cases), followed by schwannoma (12.0%), cavernous hemangioma (4.6%), and glioma (2.7%). Mean follow-up was 27.6 ± 15.1 months. CSF leak was observed in 1% (95% CI: 0.00 to 0.04, I² = 46.5%), and wound infection was observed in 3% (95% CI: 0.01 to 0.07, I²=0%). Ptosis occurred in 4% (95% CI: 0.00 to 0.14, I²=79.4%) and diplopia occurred in 6% (95% CI: 0.01 to 0.14, I²=68.9%). Medial gaze palsy occurred in 9% (95% CI: 0.04 to 0.18, I²=8.8%). Improvement in visual function was seen in 47% (95% CI: 0.22 to 0.73, I²=88.7%). Visual dysfunction occurred in 1% (95% CI: 0.00 to 0.04, I²=48.6%). Transient facial numbness occurred in 16% (95% CI: 0.09 to 0.25, I²=35.0%). Mortality was 0% (95% CI: 0.00 to 0.02, I²=28.7%). ETOA is a safe and promising technique for managing a wide range of skull base and orbital lesions. Future prospective and comparative studies are needed to refine indications and validate its long-term efficacy.

**Supplementary Information:**

The online version contains supplementary material available at 10.1007/s10143-026-04321-x.

## Introduction

The surgical management of skull base lesions remains a considerable challenge due to their complex anatomy and proximity to critical neurovascular structures. Traditional transcranial approaches, while effective, are potentially associated with significant morbidity, including retraction-related brain injury, extensive soft-tissue dissection, and suboptimal cosmetic outcomes [1]. In recent years, minimally invasive techniques have gained prominence, among which the endoscopic transorbital approach (ETOA) has emerged as a valuable alternative for selected skull base pathologies, including the anterior cranial fossa, middle cranial fossa, and Meckel’s cave [2, 3]. Foundational anatomical studies have been instrumental in defining safe and reliable surgical corridors for this approach, providing critical insights into the relationships between neurovascular structures and bony landmarks [4].

The ETOA, frequently performed through a superior eyelid crease with or without lateral orbitotomy, provides a short and direct surgical corridor, minimizing soft-tissue manipulation and often resulting in favorable cosmetic outcomes [1, 5, 6]. This route enables access to challenging skull base regions such as the sphenoid wing and adjacent skull base regions, cavernous sinus, and Meckel’s cave, often without the need for extensive craniotomy [2, 7]. The extent of orbitotomy plays a crucial role in enhancing surgical freedom; quantitative anatomical studies have demonstrated that removal of the lateral orbital rim significantly increases the angle of attack and surgical freedom for deep-seated skull base lesions, with more extensive orbitotomy yielding proportionally greater gains [8]. Clinical series have demonstrated that gross total resection (GTR) can be achieved in selected patients while maintaining low complication rates and favorable functional outcomes [2, 6, 9].

Spheno-orbital meningiomas (SOM) represent one of the most frequent indications for ETOA in skull base surgery. Several studies have shown that this approach achieves effective decompression, improves or stabilizes visual function, and reduces proptosis, with a low incidence of permanent morbidity [3, 7, 10]. Beyond meningiomas, the approach has also been applied to other skull base tumors, including schwannomas, dermoid cysts with skull base extension, and gliomas, further expanding its clinical applicability [1, 6, 11]. The versatility of the ETOA also extends to selected skull base indications [9].

Nevertheless, the ETOA has important limitations. Achieving radical resection in diffuse *en plaque* lesions, such as extensive SOM with hyperostosis, may be restricted, particularly in the presence of vascular encasement or extensive dural involvement [10]. In such cases, the therapeutic aim often shifts toward safe decompression with or without adjuvant therapies, rather than aggressive resection. When carefully indicated, outcomes appear comparable to those obtained with traditional transcranial approaches, while maintaining lower morbidity and favorable cosmetic outcomes [7, 10]. As surgical experience expands and multicenter data accumulate, the indications and applications of the ETOA are expected to be further refined, consolidating its role in contemporary skull base surgery.

Considering this background, the present study aims to systematically evaluate the spectrum of complications and morbidity associated with the ETOA in skull base lesions.

## Methods

This study was performed in line with the Preferred Reporting Items for Systematic Reviews and Meta-Analysis (PRISMA) 2020 guidelines and the recommendations of the Cochrane Collaboration Handbook for Systematic Reviews of Interventions [12, 13]. The PRISMA 2020 checklist and PRISMA Abstract checklist are provided as supplementary materials (Tables S1 and S2). The full protocol was prospectively registered in PROSPERO (CRD420251130069).

### Search strategy

We systematically searched PubMed, Embase, Scopus and Web of Science for studies until March 2026. The following search strategy was applied: (“endoscopic transorbital” OR “superior eyelid” OR “lateral orbitotomy” OR “endoscopic transorbital approach” OR “neuroendoscopic transorbital” OR “neuroendoscopic transorbital approach” OR “transorbital”) AND (“skull base” OR “meningioma” OR “orbito-cranial” OR “orbital” OR “trigeminal schwannoma” OR “glioma” OR “adenoma” OR “adenocarcinoma” OR “lacrimal gland” OR “lymphoma” OR “hemangioma” OR “cavernous hemangioma” OR “cyst” OR “epidermoid cyst” OR “dermoid cyst” OR “plasmocytoma”). Two authors (B.Z.P. and D.S.) independently screened the literature according to predefined criteria and any disagreements were resolved by a third author (R.B.A.).

### Eligibility criteria

In this meta-analysis, inclusion was limited to Randomized Controlled Trials and Observational Studies evaluating the ETOA in patients with skull base lesions. The primary outcomes of interest were complication rates, including: (1) mortality, (2) cerebrospinal fluid (CSF) leak, (3) diplopia, (4) ptosis, (5) enophthalmos, (6) infection, (7) visual acuity deterioration or improvement, (8) medial gaze palsy (MGP), and (9) transient facial numbness. Extent of resection (EOR) was a secondary outcome. Studies with fewer than five patients, as well as those employing mixed surgical approaches in the same procedure – multiportal surgery –, were excluded. Anatomical or cadaveric studies, reviews, meta-analyses, and conference abstracts were also excluded. Procedural classification (intra/extradural) was based on the primary surgical corridor described by each study. Mixed techniques were noted when applicable.

### Data extraction

During the review process, two authors (V.L.F.K. and H.P.G.) independently assessed the selected studies for data extraction to ensure accuracy and minimize bias. Any disagreements were resolved through consensus.

### Endpoints

In this study, CSF leak was defined as any fistulous communication occurring during the postoperative period. The outcome “wound infection” included any bacterial infection requiring medical or surgical management (e.g., wound washout or antibiotic therapy). Visual complications – such as ptosis, diplopia, and cranial nerve palsy – were extracted regardless of whether they were transient or permanent. For visual improvement or worsening, any partial or total change in visual function was considered based on clinical documentation. Facial numbness was evaluated through physical examination, and any degree of reduction in sensation was included in the analysis. GTR was uniformly defined across the included studies. However, some heterogeneity was noted in the definitions of subtotal resection (STR) and partial resection (PR), as these varied among studies. Lastly, recurrence was recorded when tumor regrowth was reported during follow-up, and mortality was collected regardless of the underlying cause (tumor progression or surgical complications).

### Quality assessment

Quality assessment was performed by two independent authors (G.C and R.B.A) using the Cochrane Risk of Bias in Nonrandomized Studies of Interventions (ROBINS-I) tool [14], which examines potential biases across seven domains. Discrepancies were resolved through consensus after discussion with the senior author (G.G).

### Statistical analysis

This systematic review and meta-analysis followed the Cochrane Collaboration and the PRISMA 2020 guidelines. A single proportion analysis with 95% confidence intervals (CI) was used to measure the effects. I² statistics assessed heterogeneity; I² > 35% was considered significant. Given the large number of studies investigating meningiomas, we conducted a subanalysis to identify complications specific to this condition. Statistical analysis was performed using R Studio software (version 4.2.3, R Foundation for Statistical Computing, Vienna, Austria).

## Results

### Study selection

**Fig. 1:**
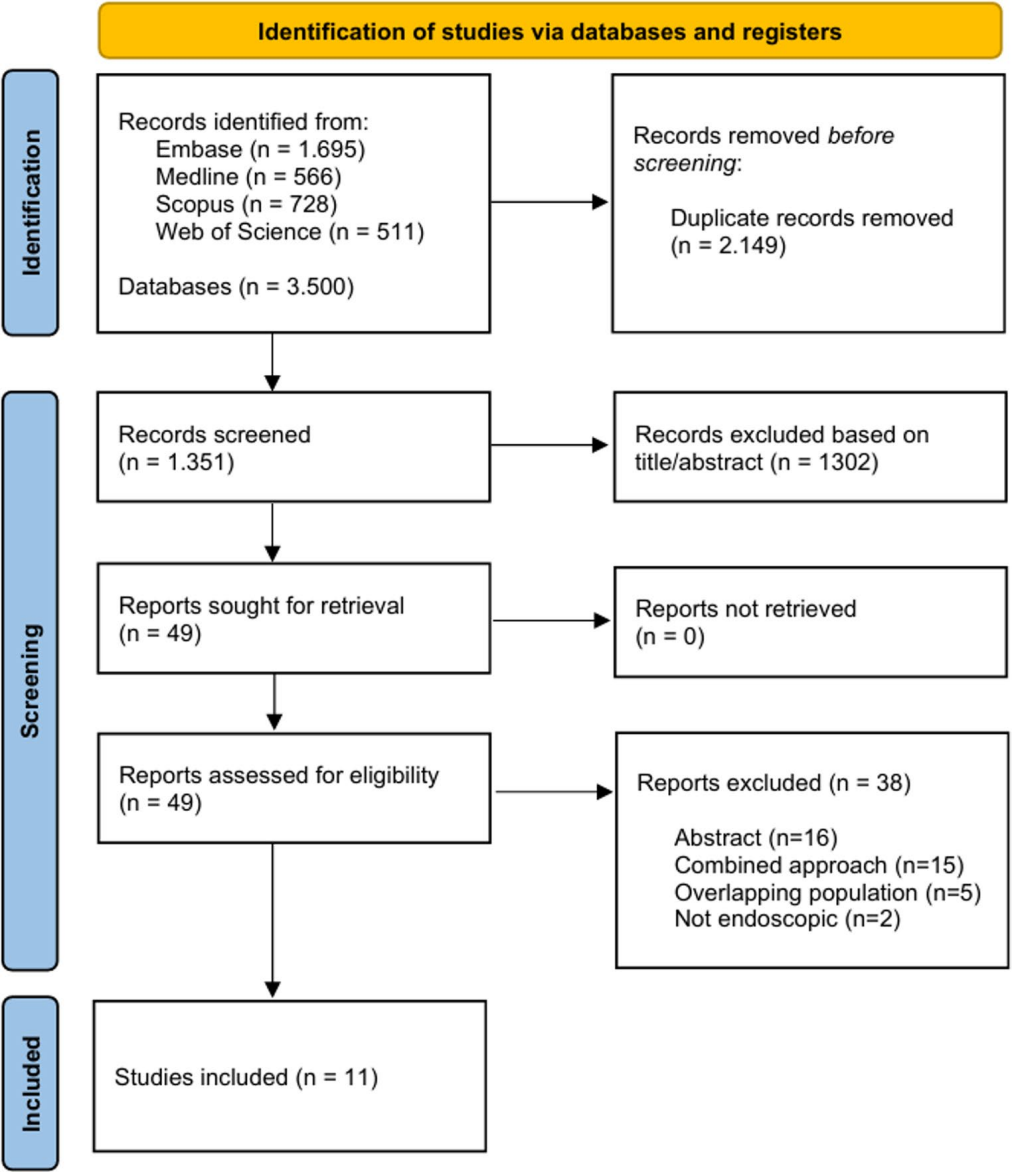
PRISMA flowchart

Initially, 3,500 articles were identified. After removing duplicates, 2,149 studies remained and their titles and abstracts were screened according to the inclusion and exclusion criteria. Of these, 49 articles were selected for full-text assessment. After a detailed evaluation, thirty-eight articles were excluded: sixteen due to being available only as conference abstracts, fifteen for employing combined approaches simultaneously with ETOA, five because they involved overlapping populations, with the smaller sample excluded, and two due to a non-endoscopic transorbital approach. Ultimately, 11 studies [1–3, 6, 7, 9, 15–19] met the eligibility criteria and were included in the final analysis (Fig. 1).

### Baseline characteristics of included studies

All 11 included studies were observational, comprising 2 prospective and 9 retrospective studies, published between 2019 and 2025. Altogether, these studies involved 269 patients who underwent ETOA for the treatment of intracranial skull base lesions. The mean patient age was 52.7 ± 3.2 years. In terms of sex distribution, the sample included 176 female (65.4%) and 93 male (34.6%) patients. Meningioma was the most frequently reported pathology (60.6%), followed by schwannoma (12.0%), cavernous hemangioma (4.6%), and glioma (2.7%), with a total of 21 pathological entities identified.

Regarding the surgical technique, lateral orbitotomy was consistently performed in 4 studies, used when necessary in 3, not employed in 1, and not specified in 3. Additionally, lateral canthotomy was performed in 2 studies, not used in 2, and not reported in 7. Dynamic orbital retraction was described in 5 studies, fixed retraction in 1, and not specified in 5. Most studies reported ETOA being performed with curative intent, while a few used it for diagnostic purposes.

Eleven studies reported mortality during procedure and follow-up, accounting for two deaths (0.7%), both for disease progression. Follow-up data were available in 10 studies, with a mean duration of 27.6 ± 15.1 months. Detailed baseline characteristics of the included studies are presented in Table 1, while procedural characteristics are summarized in Table 2.

**Table 1:** Baseline characteristics of the included studies

Study (y)	Type of Study	No. of Patients	M: F	Mean age (y)	Pathology (%)	Follow-up (mo)
Carnevale (2023) [19]	R	5	1:4	58.6	Meningioma (100)	8.9
Doo-Sik Kong (2021) [7]	P	41	5:36	52	Meningioma (100)	15.9
Golbin (2019) [16]	R	12	2:10	51	Meningioma (16.7); Pseudotumor (25); MPNST (8.3); Osteoblastoma (8.3); Metastasis (8.3); Mucocele (8.3); Others (25)	N/A
Han (2023) [2]	R	16	5:11	49	Meningioma (50); Hemangiomas (12.5); Trigeminal schwannoma (6.25); Epidermoid cyst (6.25); Glioma (6.25) ; Inflammatory hyperplasia tissue (6.25); Langerhans cell histiocytosis (6.25); Bone fibrosis hyperplasia (6.25)	26
Karimzada (2024) [15]	R	32	19:13	53.2	Meningioma (100)	16.3
Kim (2023) [9]	R	14	7:7	53.3	Meningioma (100)	16.8
Kong (2024) [17]	R	32	9:23	47.8	Meningioma (34.4); Trigeminal schwannoma (65.6)	34.7
Quillan (2025) [18]	R	35	14:21	52.5	Meningioma (31.4); Schwannoma (11.4); Fibrous tumor (11.4); Metastasis (5.7); Encephalocele (5.7); Others (34.4)	34.4
Yoo (2021) [1]	P	22	10:12	52.5	Meningioma (50); Glioma (22.7); Schwannoma (13.6); Cavernous hemangioma (4.5); Hemangiopericytoma (4.5); Lymphoma (4.5)	3.2
Zoia (2024) [6]	R	38	15:23	57	Meningioma (28.9); Cavernous haemangioma (21); Metastatic adenocarcinoma (7.8); Dermoid cyst (5.2); Epidermoid cyst (5.2); Fibroinflammatory lesion (2.6); Schwannoma (2.6); Inflammatory pseudotumor (2.6); Mucocele (2.6); Sarcoma (2.6); Metastatic squamous cell carcinoma (2.6); Mucoepidermoid carcinoma (2.6); Metastatic adenoid cystic carcinoma (2.6); Mastocytosis (2.6); B cell lymphoma (2.6); Venous varix (2.6)	55
Zoli (2023) [3]	R	22	6:16	57	Meningioma (86.5); Fibrous dysplasia (4.5); Plasmocytoma (4.5); Solitary fibrous tumor (4.5)	34

*F* female; *M* male; *mo* months; *N/A* not available; *P* prospective; *R* retrospective; *y* year

**Table 2:** Procedural characteristics of the included studies

Study (year)	Lateral Orbitotomy (Y/*N*)	Lateral Canthotomy (Y/*N*)	Fixed/dynamic orbital retraction	Intra/extradural procedure	Mean Operative Time (min)	Surgical Intent	Procedure mortality*
Carnevale (2023) [19]	N/A	Y	Dynamic	Both	N/A	Treatment	0
Doo-Sik Kong (2021) [7]	Y	N/A	N/A	Both	256	Treatment	0
Golbin (2019) [16]	N/A	N/A	N/A	Both	N/A	Treatment (50%); Diagnosis (50%)	0
Han (2023) [2]	If needed	Y	Dynamic	Both	352	Treatment (87.5%); Diagnosis (12.5%)	0
Karimzada (2024) [15]	Y	N/A	N/A	Both	256	Treatment	0
Kim (2023) [9]	Y	N/A	N/A	Extradural	N/A	Treatment	0
Kong (2024) [17]	If needed	N/A	N/A	Extradural (transcavernous)	N/A	Treatment	0
Quillan (2025) [18]	Y	N/A	Dynamic	Both	342	Treatment	0
Yoo (2021) [1]	If needed	N	Fixed	Intradural	N/A	Treatment	0
Zoia (2024) [6]	N/A	N/A	Dynamic	Both	124	Treatment (92.1); Diagnosis (7.9)	0
Zoli (2023) [3]	N	N	Dynamic	Both	N/A	Treatment	0

*min* minutes; *N* no; *N/A* not available; *Y* yes

*Only deaths directly related to the surgical procedure were considered in this analysis. Mortality resulting from other causes or disease progression during follow-up was not included in the table

### Quality assessment

Methodological quality of the eleven included non-randomized studies was assessed using the ROBINS-I tool, which evaluates seven domains of bias. The risk of bias for each domain was rated as low, moderate, or serious, and the overall risk for each study was determined based on the cumulative assessment across all domains.

Four studies were judged to have a moderate risk of bias, whereas seven were rated as having a serious risk of bias. The main concerns were related to confounding factors, participant selection, outcome measurement, and selective reporting of results. Moreover, the exclusively observational design of all included studies introduces an inherent source of bias that should be taken into account. The corresponding assessments are visually summarized in the Traffic Light Plot and Summary Plot (Figures S1 and S2) is provided in the Supplementary Material, offering an overview of the domains evaluated.

### Pooled analysis

#### Postoperative cerebrospinal fluid leak

**Fig. 2:**
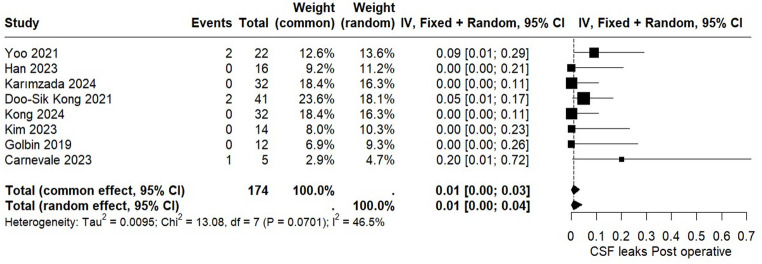
Forest plot for CSF leak postoperative

The incidence of postoperative CSF leak following the ETOA was assessed in eight studies encompassing a total of 174 patients. The pooled analysis demonstrated a CSF leak rate of 1% (95% CI: 0.00 to 0.04). The I² value of 46.5% indicates moderate heterogeneity among the included studies, most related to studies with small sample size. Further details are presented in Fig. 2.

#### Wound infection

**Fig. 3:**
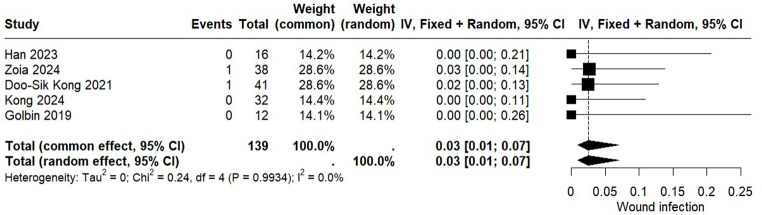
Forest plot for wound infection postoperative

The incidence of postoperative wound infection following the ETOA was assessed in three studies encompassing a total of 139 patients. The pooled analysis demonstrated a wound infection rate of 3% (95% CI: 0.01 to 0.07). The I² value of 0.0% demonstrates no heterogeneity among the included studies, indicating consistent findings across the analyses. Additional details are provided in Fig. 3.

#### Ophthalmologic outcomes

**Fig. 4:**
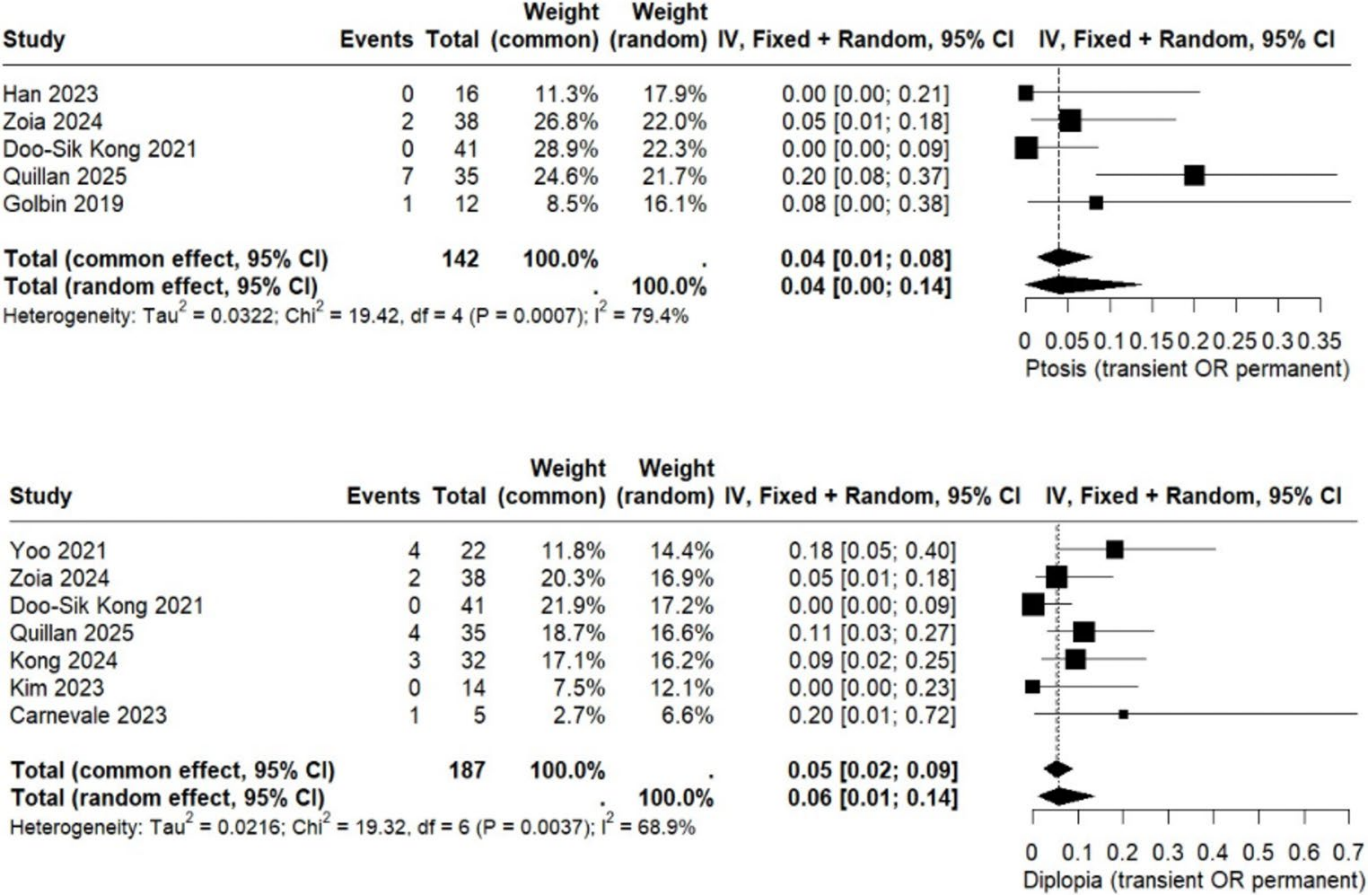
Forest plot ptosis and diplopia postoperative

The first ophthalmologic outcome analyzed was ptosis, reported in five studies comprising a total of 142 patients. The pooled analysis revealed a postoperative ptosis rate of 4% (95% CI: 0.00 to 0.14). The I² value of 79.4% indicates high heterogeneity. Additional details are presented in Fig. 4. Furthermore, diplopia was reported in seven studies including a total of 187 patients. The pooled postoperative rate of diplopia was 6% (95% CI: 0.01 to 0.14). Further details are provided in Fig. 4.

Additionally, postoperative rates of MGP were analyzed across five studies comprising a total of 120 patients. The pooled rate of MGP was 9% (95% CI: 0.04 to 0.18), with low heterogeneity (I² = 8.8%). Further details are available in the Supplementary Material Figures S4 .

**Fig. 5:**
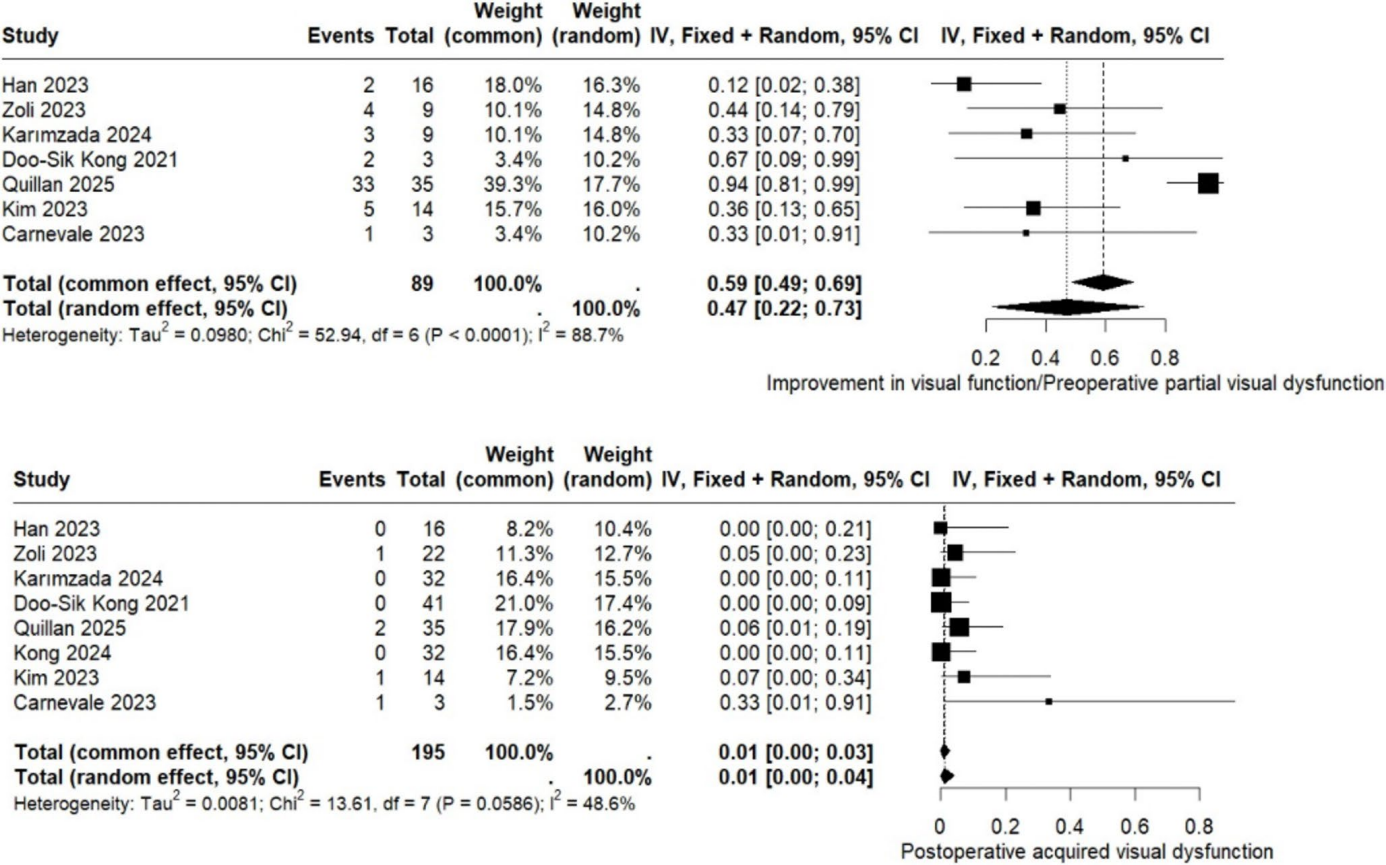
Forest plot for visual improvement and visual dysfunction postoperative

Improvement in postoperative visual function was assessed in eight studies including 89 patients. The pooled analysis revealed a visual improvement rate of 47% (95% CI: 0.22 to 0.73), accompanied by a high degree of heterogeneity (I² = 88.7%). Additional results are presented in Fig. 5. The Baujat plot identified Quillan et al. (2025) [18] as a major contributor to the overall heterogeneity and effect estimate; the leave-one-out analysis demonstrated that excluding Quillan et al. (2025) [18] reduced heterogeneity to 14%, yielding a pooled visual improvement rate of 32% (95% CI: 0.18 to 0.47). Supplementary Material Figures S5 provides further details.

Finally, postoperative visual dysfunction was evaluated in eight studies encompassing 195 patients. The pooled analysis demonstrated a rate of 1% (95% CI: 0.00 to 0.04), with an I² value of 48.6%, indicating moderate heterogeneity (Fig. 5).

#### Transient facial numbness

**Fig. 6:**
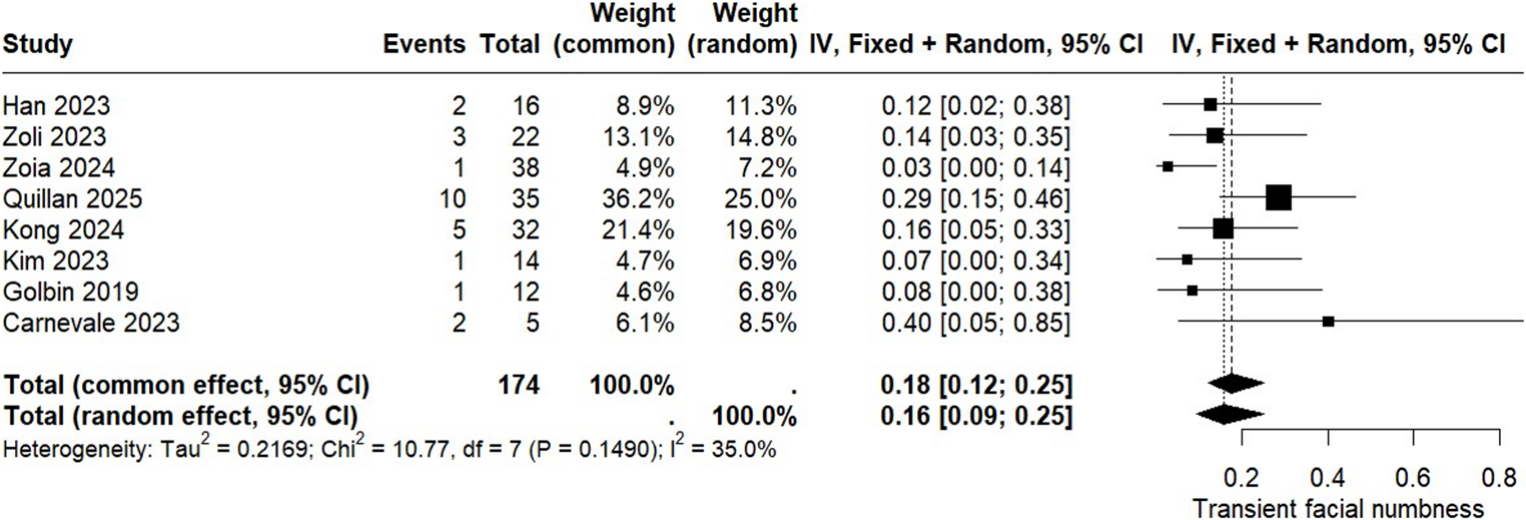
Forest plot for transient facial numbness during postoperative

Transient facial numbness was evaluated in eight studies including a total of 174 patients. The pooled rate of this outcome was 16% (95% CI: 0.09 to 0.25), with an acceptable level of heterogeneity (I² = 35.0%). The corresponding Forest Plot is presented in Fig. 6.

#### Extent of resection

The EOR was categorized into three groups and analyzed separately: GTR, STR, and PR. In this analysis, we excluded three cases from the series by Han et al. (2023) [2], as these procedures were performed for biopsy purposes. All outcomes exhibited high heterogeneity, primarily attributed to the variability of pathologies included in our analysis. These results, presented in Supplementary Figure S6, should therefore be interpreted with caution.

GTR was evaluated in ten studies comprising a total of 254 patients. It was achieved in 56% of cases (95% CI: 0.39 to 0.72) under a random-effects model, with substantial heterogeneity (I² = 76.6%) that could not be mitigated in the sensitivity analysis. STR was reported in eleven studies involving 260 patients and was achieved in 21% of cases (95% CI: 0.09 to 0.36), also under a random-effects model. Heterogeneity remained high (I² = 85.6%) and persisted despite sensitivity testing. Finally, PR was assessed in eight studies including 161 patients, being achieved in only three cases, resulting in a pooled PR rate of 1% (95% CI: 0.00 to 0.06) under a random-effects model, with heterogeneity of I² = 58.3%.

#### Recurrence and mortality

Recurrence rate was analyzed in eight studies encompassing 151 patients. The pooled recurrence rate was 1% (95% CI: 0.00 to 0.08), with substantial heterogeneity (I² = 74.6%). Further details are shown in Supplementary Fig. 7. The Baujat plot indicated that Zoli et al. (2023) [3] contributed significantly to both the overall heterogeneity and the pooled estimate. The leave-one-out analysis demonstrated that excluding this study reduced the pooled recurrence rate to 0% (95% CI: 0.00 to 0.04). These results are presented in Supplementary Material (Figures S8 and S9). Nevertheless, this outcome should also be interpreted with caution, given the heterogeneity of the underlying pathologies included in our analysis, which naturally exhibit distinct biological behavior and prognosis.

The final outcome assessed was mortality, reported in eight studies comprising 172 patients. The pooled mortality rate was 0% (95% CI: 0.00 to 0.02), with low heterogeneity (I² = 28.7%). Additional details are provided in Supplementary Fig. 7. It is noteworthy that the two deaths observed during follow-up were attributable to tumor progression rather than postoperative complications.

#### Subanalysis for meningiomas

Finally, we performed a subanalysis focusing specifically on outcomes reported for meningiomas, the most prevalent pathology in our review. Only studies that included exclusively meningioma cases were considered, yielding four eligible studies. All results are summarized in the Supplementary Material (Figures S10 to S15).

Postoperative CSF leak was reported in 92 patients, with a pooled rate of 6% (95% CI: 0.02 to 0.14) and low heterogeneity (I² = 0.0%). These results did not differ substantially from the overall analysis. Improvement in visual function was reported for 29 patients, with a pooled rate of 38% (95% CI: 0.22 to 0.57) and low heterogeneity (I² = 0.0%), again reflecting trends similar to the general analysis. Visual dysfunction was reported for 90 patients, with two cases observed, resulting in a pooled rate of 5% (95% CI: 0.01 to 0.23), with moderate heterogeneity (I² = 42.6%), consistent with the overall sample.

GTR was analyzed in 92 patients, with a pooled rate of 41% (95% CI: 0.07 to 0.87) and heterogeneity (I² = 73.3%). STR was assessed in 92 patients, yielding a pooled rate of 18% (95% CI: 0.05 to 0.47) and similarly high heterogeneity (I² = 80.3%). Given the considerable variability in meningioma characteristics between these studies, these results should be interpreted with caution. Finally, no deaths were reported during the procedure or follow-up (0%; 95% CI: 0.01 to 0.10; I² = 0%).

## Discussion

The primary objective of this systematic review and meta-analysis was to evaluate complications of the endoscopic transorbital approach (ETOA) for skull base pathologies. Analyzing eleven observational studies comprising 269 patients, we found low postoperative complication rates: CSF leak (1%), wound infection (3%), ptosis (4%), diplopia (6%), and medial gaze palsy (9%). Visual improvement occurred in 47% of patients, with visual deterioration in only 1%. Transient facial numbness was reported in 16%. Gross-total resection (GTR) was achieved in 56% of procedures (notable heterogeneity), and subtotal resection (STR) in 21%. Recurrence and mortality rates during follow-up were 1% and 0%, respectively.

Our findings align with and extend previous systematic reviews. Vural et al. (2021) summarized the surgical anatomy and clinical applications of transorbital approaches based on 42 studies (193 patients) [20]. Foundational anatomical studies have since refined safe surgical corridors, providing critical insights into neurovascular relationships [4]. Quantitative anatomical research has demonstrated that lateral orbital rim removal significantly increases surgical freedom for deep-seated skull base lesions [8]. While previous work provided foundational anatomical and qualitative synthesis, our study offers novel contributions: (1) a quantitative meta-analysis with pooled complication rates from 269 patients across 11 studies; (2) focused assessment of ophthalmologic outcomes; (3) a meningioma-specific subanalysis; and (4) evaluation of heterogeneity sources through sensitivity and leave-one-out analyses. Thus, our study provides the first robust quantitative synthesis of safety and functional outcomes for ETOA.

The interpretive focus of our findings lies on safety rather than cytoreduction. Given the 21 distinct pathologies in our study, the pooled GTR estimate is inherently unstable. A recent systematic review proposed location-specific subgroup analysis, demonstrating significant differences in complication rates across anatomical compartments [21]. However, such stratification was not feasible in our dataset due to inconsistent reporting of lesion location and limited events within subgroups. Our analysis therefore prioritizes a technique-oriented perspective, focusing on complications intrinsic to the transorbital corridor. Contemporary series support this approach: spheno-orbital meningioma cohorts show uniform functional gains with minimal morbidity even when GTR is limited [3]; multicenter experience confirms that tumor morphology and corridor optimization are the true determinants of resectability [7]; and broader middle-fossa cohorts illustrate real-world variance without undermining the low permanent-morbidity profile [1].

Our pooled estimates define a reproducible complication profile: CSF leak (1%), wound infection (3%), ptosis (4%), diplopia (6%), medial gaze palsy (9%), visual worsening (1%), transient facial numbness (16%), and mortality (0%). Sensitivity analyses confirmed that dispersion in oculomotor events is largely driven by a few middle-fossa series reporting transient deficits [1], whereas cohorts emphasizing lateral-rim osteotomy and meticulous closure frequently report zero CSF leaks and no permanent visual decline [2]. This favorable safety profile is further corroborated by a recent meta-analysis by Corvino et al. 2025, which identified a pooled CSF leak rate of 2.5% across 240 cases, reinforcing the reliability of skull base reconstruction and the inherently low risk of postoperative CSF leakage following ETOA [22]. Furthermore, case selection should prioritize functional goals (vision/proptosis) over GTR for en-plaque tumors; corridor optimization should minimize traction-related neuropathies; and standardized ophthalmic outcome reporting should accompany EOR in future studies.

Beyond global safety trends, specific patterns emerged for ocular and sensory morbidity. Oculomotor deficits diplopia, ptosis, and MGP occurred in 4–9% of patients. These events were predominantly transient and often associated with extended extradural or interdural dissection around the superior orbital fissure or cavernous sinus. This suggests that ocular motility deficits in ETOA are typically traction- or edema-related rather than due to direct nerve injury, consistent with anatomical observations that cranial nerves III, IV, and VI lie at the edge of the working corridor. The low rate of permanent deficits across modern series [2, 3] indicates that meticulous interdural plane preservation and dynamic (rather than fixed) orbital retraction are key to preventing these complications.

Our findings highlight an evolution in the balance between surgical aggressiveness and morbidity compared to traditional transcranial literature. Large historical series (e.g., Ringel et al., Terrier et al.) showed that while transcranial surgery improves proptosis and visual function, it carries high rates of permanent cranial nerve deficits (up to 30%), CSF leaks, wound infections, and even mortality [23, 24]. This led to the long-standing conclusion that symptom-oriented decompression, not radical resection, should guide management in some situations. In contrast, our pooled ETOA analysis achieves similar functional goals (optic decompression and visual preservation) with markedly lower morbidity: CSF leak/infection ~ 1%, low rates of permanent oculomotor deficits, visual deterioration ~ 1%, and zero procedure-related mortality. Thus, while transcranial approaches remain indispensable for large, multicompartmental tumors, ETOA offers a low-morbidity alternative for selected patients, achieving the same symptom-oriented objectives with a superior safety profile.

Clinically, these data position the transorbital corridor as a complementary, low-morbidity route whose value is greatest when goals are diagnostic access, decompression, or targeted debulking in anatomically constrained territory (lateral cavernous wall, optic canal, and anterior skull base compartments). Conversely, selected skull base lesions without vascular encasement are scenarios in which ETOA can legitimately pursue curative or near-total resection while maintaining a favorable safety profile [7]. Multidisciplinary execution (neurosurgery + oculoplastics), lateral rim osteotomy when indicated, interdural cavernous exposure, and meticulous layered closure are practical levers that keep permanent morbidity rare while shortening length of stay and preserving cosmesis.

### Limitations

Our study’s limitations reflect the current state of evidence in this field. Although we restricted analysis to skull base lesions to improve methodological consistency, the inclusion of multiple pathologies still introduces clinical and statistical heterogeneity. Most included studies are observational, with single-arm proportions, short and variable follow-up, and non-uniform definitions for EOR and ophthalmic outcomes, limiting interpretability of pooled complication rates. Specific oculomotor outcomes were inconsistently reported, precluding dedicated analysis despite their anatomical relevance. Even with leave-one-out and Baujat analyses, residual heterogeneity persists, and pooled GTR estimates should be interpreted as context-dependent rather than approach-defining. Reporting bias toward favorable cases and inconsistent documentation of complications (temporary vs. permanent) further limit cross-study comparability. These constraints support cautious interpretation of cytoreductive outcomes and reinforce the value of evaluating ETOA primarily through consistent safety endpoints (CSF leak, infection, visual preservation). Publication bias was assessed using funnel plots for outcomes with sufficient studies. However, the small number of included studies (*n* = 11) limits funnel plot reliability and precludes formal asymmetry testing (e.g., Egger’s test). Although visual inspection did not suggest significant asymmetry for most outcomes, publication bias cannot be excluded, particularly given the predominance of studies reporting favorable outcomes.

Future work should consolidate pathology-specific core outcome sets for skull base transorbital surgery, including standardized BCVA and perimetry, as well as harmonized definitions of complications (temporary vs. permanent). These should be embedded in prospective registries with long-term follow-up (≥ 5 years). Comparative studies by indication should integrate volumetric EOR with patient-reported outcomes, including vision-related quality of life. Additionally, learning-curve analyses and cost-effectiveness studies may help define how surgical experience, corridor optimization, and reconstruction techniques influence complication rates and long-term outcomes.

## Conclusion

In conclusion, this systematic review and meta-analysis suggest that the ETOA is a safe and effective technique for selected skull base lesions, with low complication rates, favorable visual preservation, and no reported mortality. However, given the residual heterogeneity across studies, these findings should be interpreted with caution. Rather than prioritizing maximal resection, ETOA appears to achieve favorable functional outcomes with minimal morbidity. It should therefore be considered a complementary minimally invasive corridor for selected skull base pathologies. Further prospective studies are needed to better define its indications and role in contemporary skull base surgery.

## Electronic Supplementary Material

Below is the link to the electronic supplementary material.

Supplementary Material 1

## Data Availability

No datasets were generated or analysed during the current study.
