# Supplementary material for: Safety profile and surgical outcomes of the endoscopic transorbital approach as a skull base surgical corridor: a systematic review and meta-analysis
Source: Neurosurg Rev. 2026 May 13;49(1):403. doi: 10.1007/s10143-026-04321-x (PMC13167805; doi:10.1007/s10143-026-04321-x)

**Endoscopic Transorbital Approach for Skull Base and Orbital Pathologies: A Systematic Review and Meta-Analysis**

# Supplementary Material

# Table of Contents

**Table S1: PRISMA checklist** ........................................ 3
**Table S1: PRISMA Abstract checklist** ........................................ 5
**Figure S1: Traffic light plot for quality assessment** ........................................ 6
**Figure S2: Summary plot for quality assessment**........................................ 7
**Figure S3: Forest plot for medial gaze palsy**........................................ 7
**Figure S4: Baujat plot for improvement in visual function**........................................ 8
**Figure S5: Leave-one-out analysis for improvement in visual function** ........................................ 9
**Figure S6: Forest plot for extent of resection**…………….......................................... 10
**Figure S7: Forest Plot for recurrence during follow-up and mortality**…........................................ 11
**Figure S8: Baujat plot for recurrence rates during follow-up**........................................ 12
**Figure S9: Leave-one-out analysis for recurrence rates during follow-up**........................................13
**Figure S10: Forest plot for CSF leak in the treatment of meningiomas** ........................................ 13
**Figure S11: Forest plot for improvement in visual function in the treatment of meningiomas**........... 13
**Figure S12: Forest plot for visual dysfunction in the treatment of meningiomas** ................................. 14
**Figure S13: Forest plot for gross-total resection (GTR) in the treatment of meningiomas**…..….... 14
**Figure S14: Forest plot for subtotal resection (STR) in the treatment of meningiomas.** ....................14
**Figure S15: Forest plot for mortality in the treatment of meningiomas**……………………............. 15
**Figure S16: Funnel plot CSF leak Post Operative** ……………………………………………15
**Figure S17: Funnel plot Wound infection** ………………………………………………….. 16
**Figure S18: Funnel plot Ptosis**………………………………………………………………..16
**Figure S19: Funnel plot Diplopia**…………………………………………………………… 17
**Figure S20: Funnel plot Visual improvement**……………………………………………… 17
**Figure S21: Funnel plot Visual dysfunction** ………………………………………………….. 18
**Figure S22: Funnel plot Transient facial numbness**…………………………………………. 18
**Figure S23: Funnel plot Gross-total resection (GTR)**........................................ 19
**Figure S24: Funnel plot Subtotal resection (STR)**........................................ 19
**Figure S25: Funnel plot Subtotal Partial resection (PR)**........................................ 20
**Figure S26:Funnel plot Recurrence,,**………………………………………………………….. 20
**Figure S27:Funnel plot Mortality**…………………………………………………………… 21
**Figure S28:Funnel plot Enophthalmos**……………………………………………………… 21

Table S1: PRISMA checklist

| Section and Topic | Item # | Checklist item | Location where item is reported |
|---|---|---|---|
| **TITLE** | | | |
| Title | 1 | Identify the report as a systematic review. | Title Page |
| **ABSTRACT** | | | |
| Abstract | 2 | See the PRISMA 2020 for Abstracts checklist. | Title Page |
| **INTRODUCTION** | | | |
| Rationale | 3 | Describe the rationale for the review in the context of existing knowledge. | p. 3-4 |
| Objectives | 4 | Provide an explicit statement of the objective(s) or question(s) the review addresses. | p. 4 |
| **METHODS** | | | |
| Eligibility criteria | 5 | Specify the inclusion and exclusion criteria for the review and how studies were grouped for the syntheses. | p. 4 |
| Information sources | 6 | Specify all databases, registers, websites, organisations, reference lists and other sources searched or consulted to identify studies. Specify the date when each source was last searched or consulted. | p. 4 |
| Search strategy | 7 | Present the full search strategies for all databases, registers and websites, including any filters and limits used. | p. 4 |
| Selection process | 8 | Specify the methods used to decide whether a study met the inclusion criteria of the review, including how many reviewers screened each record and each report retrieved, whether they worked independently, and if applicable, details of automation tools used in the process. | p. 4 |
| Data collection process | 9 | Specify the methods used to collect data from reports, including how many reviewers collected data from each report, whether they worked independently, any processes for obtaining or confirming data from study investigators, and if applicable, details of automation tools used in the process. | p. 5 |
| Data items | 10a | List and define all outcomes for which data were sought. Specify whether all results that were compatible with each outcome domain in each study were sought (e.g. for all measures, time points, analyses), and if not, the methods used to decide which results to collect. | p. 5 |
| | 10b | List and define all other variables for which data were sought (e.g. participant and intervention characteristics, funding sources). Describe any assumptions made about any missing or unclear information. | p. 5 ; p. 7-8 |
| Study risk of bias assessment | 11 | Specify the methods used to assess risk of bias in the included studies, including details of the tool(s) used, how many reviewers assessed each study and whether they worked independently, and if applicable, details of automation tools used in the process. | p. 5 |
| Effect measures | 12 | Specify for each outcome the effect measure(s) (e.g. risk ratio, mean difference) used in the synthesis or presentation of results. | p. 6 |
| Synthesis methods | 13a | Describe the processes used to decide which studies were eligible for each synthesis (e.g. tabulating the study intervention characteristics and comparing against the planned groups for each synthesis (item #5)). | p. 4; p. 6; p. 6-7 |
| | 13b | Describe any methods required to prepare the data for presentation or synthesis, such as handling of missing summary statistics, or data conversions. | p. 5; p. 6 |

| | | | |
|---|---|---|---|
| | 13c | Describe any methods used to tabulate or visually display results of individual studies and syntheses. | p. 9-12 |
| | 13d | Describe any methods used to synthesize results and provide a rationale for the choice(s). If meta- analysis was performed, describe the model(s), method(s) to identify the presence and extent of statistical heterogeneity, and software package(s) used. | p. 6 |
| | 13e | Describe any methods used to explore possible causes of heterogeneity among study results (e.g. subgroup analysis, meta-regression). | p. 6 |
| | 13f | Describe any sensitivity analyses conducted to assess robustness of the synthesized results. | p. 10-11 |
| Reporting bias assessment | 14 | Describe any methods used to assess risk of bias due to missing results in a synthesis (arising from reporting biases). | p. 5; p. 8 |
| Certainty assessment | 15 | Describe any methods used to assess certainty (or confidence) in the body of evidence for an outcome. | p. 16-17 |
| **RESULTS** | | | |
| Study selection | 16a | Describe the results of the search and selection process, from the number of records identified in the search to the number of studies included in the review, ideally using a flow diagram. | p. 6-7 |
| | 16b | Cite studies that might appear to meet the inclusion criteria, but which were excluded, and explain why they were excluded. | p. 6-7 |
| Study characteristics | 17 | Cite each included study and present its characteristics. | p. 7-8 |
| Risk of bias in studies | 18 | Present assessments of risk of bias for each included study. | p. 8 |
| Results of individual studies | 19 | For all outcomes, present, for each study: (a) summary statistics for each group (where appropriate) and (b) an effect estimate and its precision (e.g. confidence/credible interval), ideally using structured tables or plots. | p. 9-12 |
| Results of syntheses | 20a | For each synthesis, briefly summarise the characteristics and risk of bias among contributing studies. | p. 9-12 |
| | 20b | Present results of all statistical syntheses conducted. If meta-analysis was done, present for each the summary estimate and its precision (e.g. confidence/credible interval) and measures of statistical heterogeneity. If comparing groups, describe the direction of the effect. | p. 9-12 |
| | 20c | Present results of all investigations of possible causes of heterogeneity among study results. | p. 10 |
| | 20d | Present results of all sensitivity analyses conducted to assess the robustness of the synthesized results. | p. 10-11 |
| Reporting biases | 21 | Present assessments of risk of bias due to missing results (arising from reporting biases) for each synthesis assessed. | p. 8 |
| Certainty of evidence | 22 | Present assessments of certainty (or confidence) in the body of evidence for each outcome assessed. | p. 16-17 |
| **DISCUSSION** | | | |
| Discussion | 23a | Provide a general interpretation of the results in the context of other evidence. | p. 13-16 |
| | 23b | Discuss any limitations of the evidence included in the review. | p. 16-17 |

| | | | |
|---|---|---|---|
| | 23c | Discuss any limitations of the review processes used. | p. 16-17 |
| | 23d | Discuss implications of the results for practice, policy, and future research. | p. 15-17 |
| **OTHER INFORMATION** | | | |
| Registration and protocol | 24a | Provide registration information for the review, including register name and registration number, or state that the review was not registered. | p. 4 |
| | 24b | Indicate where the review protocol can be accessed, or state that a protocol was not prepared. | p. 4 |
| | 24c | Describe and explain any amendments to information provided at registration or in the protocol. | Not applicable |
| Support | 25 | Describe sources of financial or non-financial support for the review, and the role of the funders or sponsors in the review. | p. 17 |
| Competing interests | 26 | Declare any competing interests of review authors. | p. 17 |
| Availability of data, code and other materials | 27 | Report which of the following are publicly available and where they can be found: template data collection forms; data extracted from included studies; data used for all analyses; analytic code; any other materials used in the review. | p. 4 |

Page MJ, McKenzie JE, Bossuyt PM, Boutron I, Hoffmann TC, Mulrow CD, et al. The PRISMA 2020 statement: an updated guideline f or reporting systematic reviews. BMJ 2021;372:n71.doi: 10.1136/bmj.n71. This work is licensed under CC BY 4.0. To view a copy of this license, visit https://creativecommons.org/licenses/by/4.0/

## Table S2: PRISMA Abstract checklist

| Section and Topic | Item # | Checklist item | Reported (Yes/No) |
|---|---|---|---|
| **TITLE** | | | |
| Title | 1 | Identify the report as a systematic review. | Yes |
| **BACKGROUND** | | | |
| Objectives | 2 | Provide an explicit statement of the main objective(s) or question(s) the review addresses. | _Yes |
| **METHODS** | | | |
| Eligibility criteria | 3 | Specify the inclusion and exclusion criteria for the review. | Yes |
| Information sources | 4 | Specify the information sources (e.g. databases, registers) used to identify studies and the date when each was last searched. | Yes |
| Risk of bias | 5 | Specify the methods used to assess risk of bias in the included studies. | Yes |
| Synthesis of results | 6 | Specify the methods used to present and synthesise results. | Yes |
| **RESULTS** | | | |
| Included studies | 7 | Give the total number of included studies and participants and summarise relevant characteristics of studies. | Yes |
| Synthesis of results | 8 | Present results for main outcomes, preferably indicating the number of included studies and participants for each. If meta-analysis was done, report the summary estimate and confidence/credible interval. If comparing groups, indicate the direction of the effect (i.e. which group is favoured). | Yes |
| **DISCUSSION** | | | |

| | | | |
|---|---|---|---|
| Limitations of evidence | 9 | Provide a brief summary of the limitations of the evidence included in the review (e.g. study risk of bias, inconsistency and imprecision). | Yes |
| Interpretation | 10 | Provide a general interpretation of the results and important implications. | Yes |
| **OTHER** | | | |
| Funding | 11 | Specify the primary source of funding for the review. | Yes |
| Registration | 12 | Provide the register name and registration number. | Yes |

Page MJ, McKenzie JE, Bossuyt PM, Boutron I, Hoffmann TC, Mulrow CD, et al. The PRISMA 2020 statement: an updated guideline for reporting systematic reviews. BMJ 2021;372:n71.doi: 10.1136/bmj.n71. This work is licensed under CC BY 4.0. To view a copy of this license, visit https://creativecommons.org/licenses/by/4.0/

## Figure S1: Traffic light plot for quality assessment

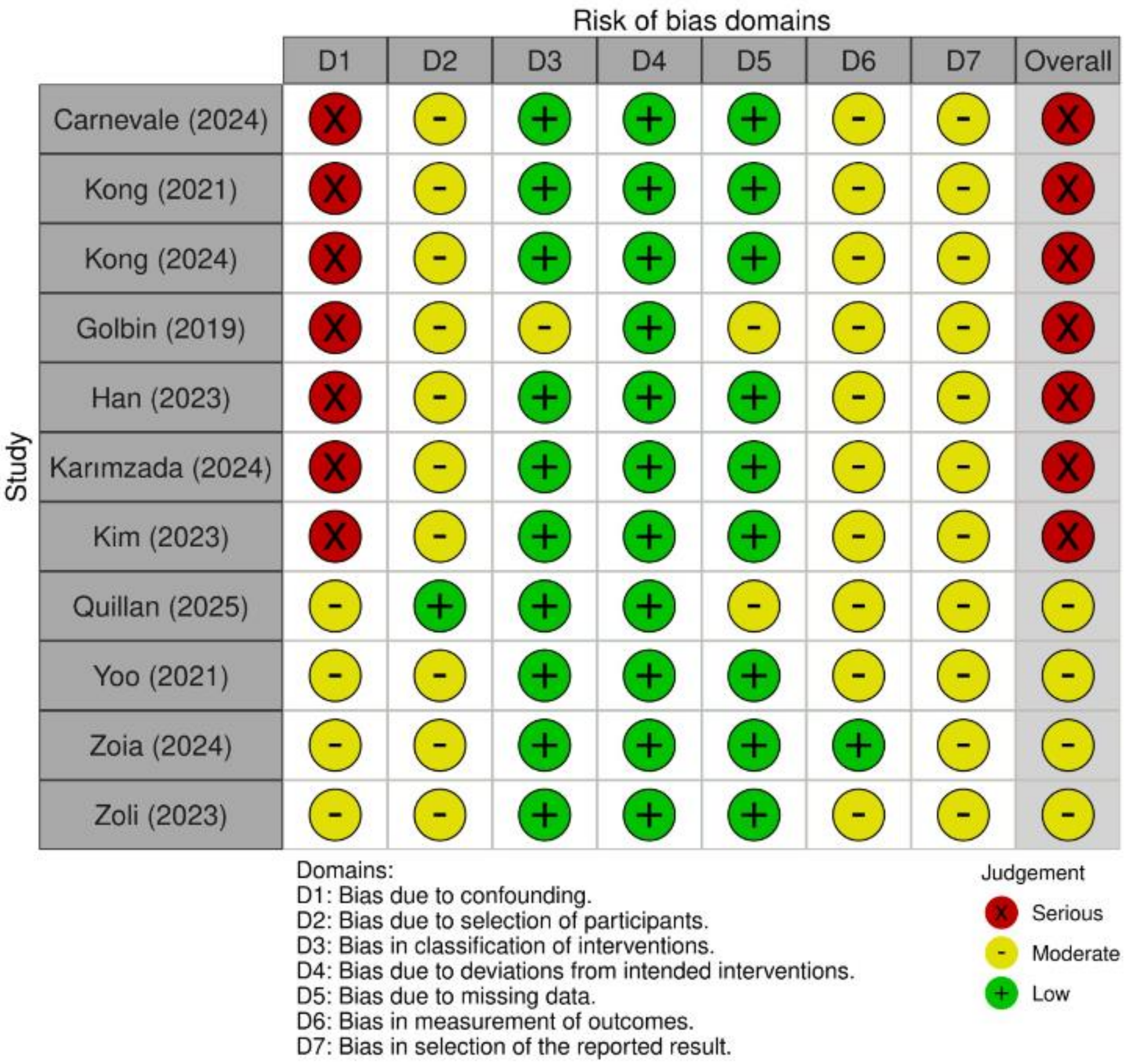

## Figure S2: Summary plot for quality assessment

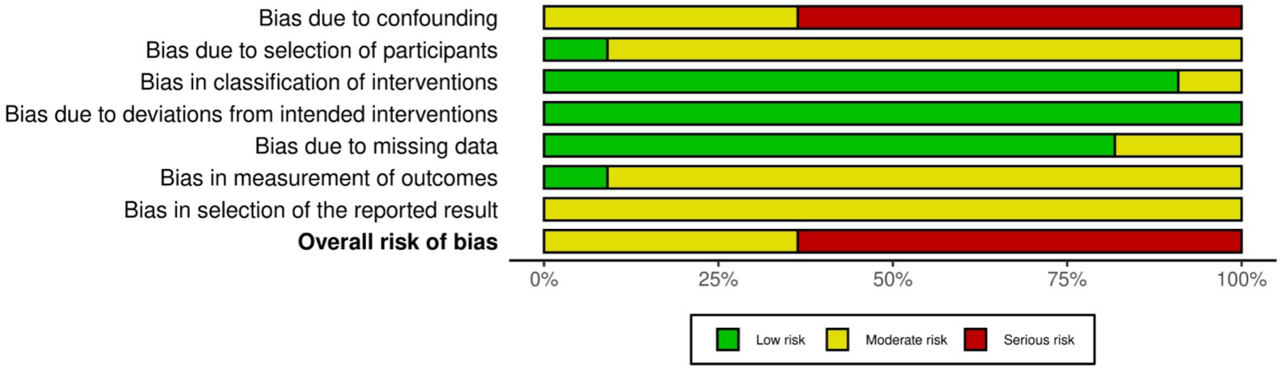


## Figure S3: Forest plot for medial gaze palsy

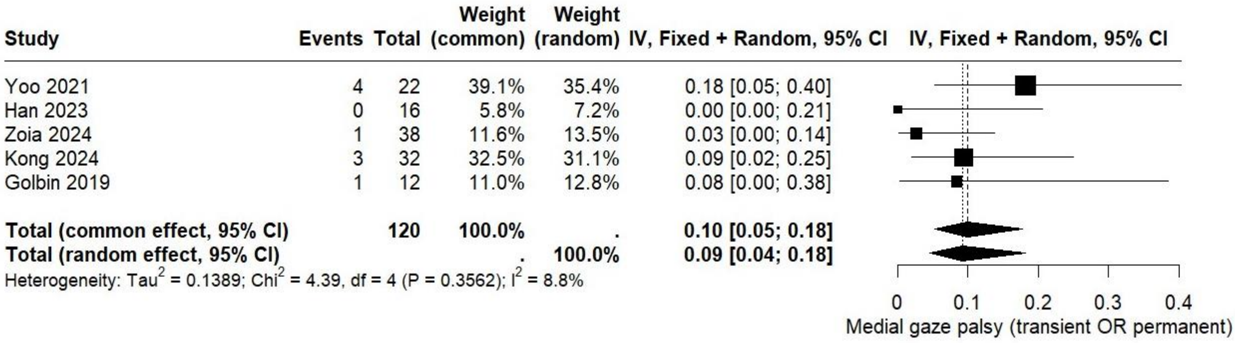

Figure S4: Baujat plot for improvement in visual function

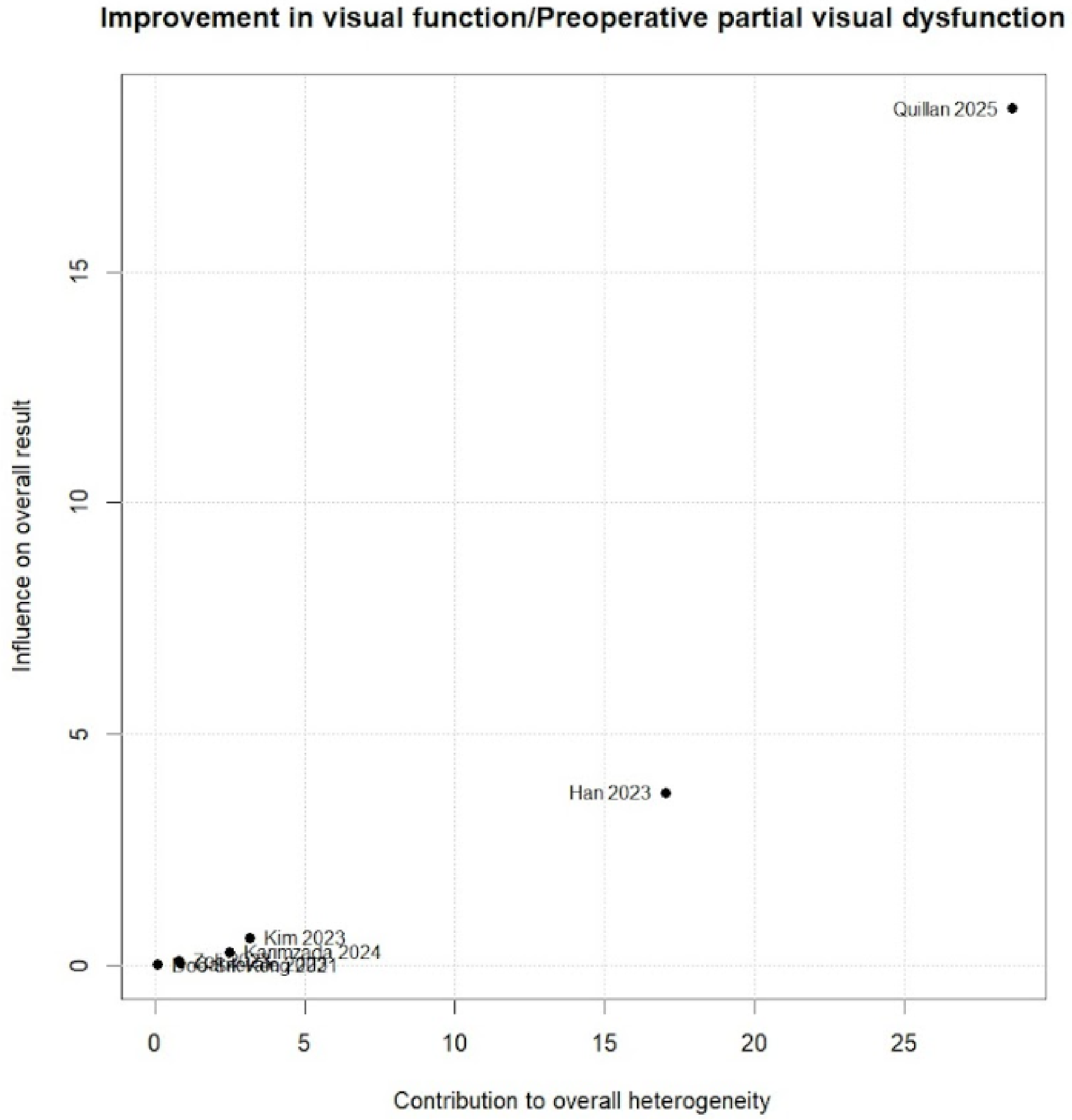

Figure S5: Leave-one-out analysis for improvement in visual function

| Study | P-value | Tau2 | Tau | I2 | IV, Random, 95% CI |
|---|---|---|---|---|---|
| Omitting Han 2023 | . | 0.0805 | 0.2837 | 84.4% | 0.55 [0.29; 0.80] |
| Omitting Zoli 2023 | . | 0.1181 | 0.3436 | 90.4% | 0.47 [0.19; 0.77] |
| Omitting Karımzada 2024 | . | 0.1141 | 0.3377 | 90.0% | 0.49 [0.21; 0.78] |
| Omitting Doo-Sik Kong 2021 | . | 0.1094 | 0.3307 | 90.5% | 0.45 [0.18; 0.73] |
| Omitting Quillan 2025 | . | 0.0090 | 0.0950 | 14.1% | 0.32 [0.18; 0.47] |
| Omitting Kim 2023 | . | 0.1163 | 0.3411 | 89.8% | 0.49 [0.20; 0.78] |
| Omitting Carnevale 2023 | . | 0.1105 | 0.3324 | 90.4% | 0.49 [0.21; 0.77] |
| **Total (95% CI)** | **.** | **0.0980** | **0.3131** | **88.7%** | **0.47 [0.22; 0.73]** |

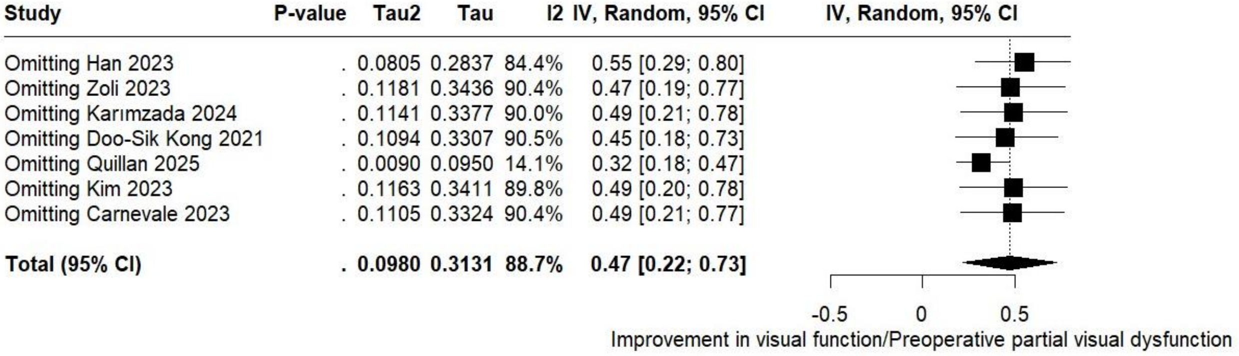

# Figure S6: Forest plot for extent of resection

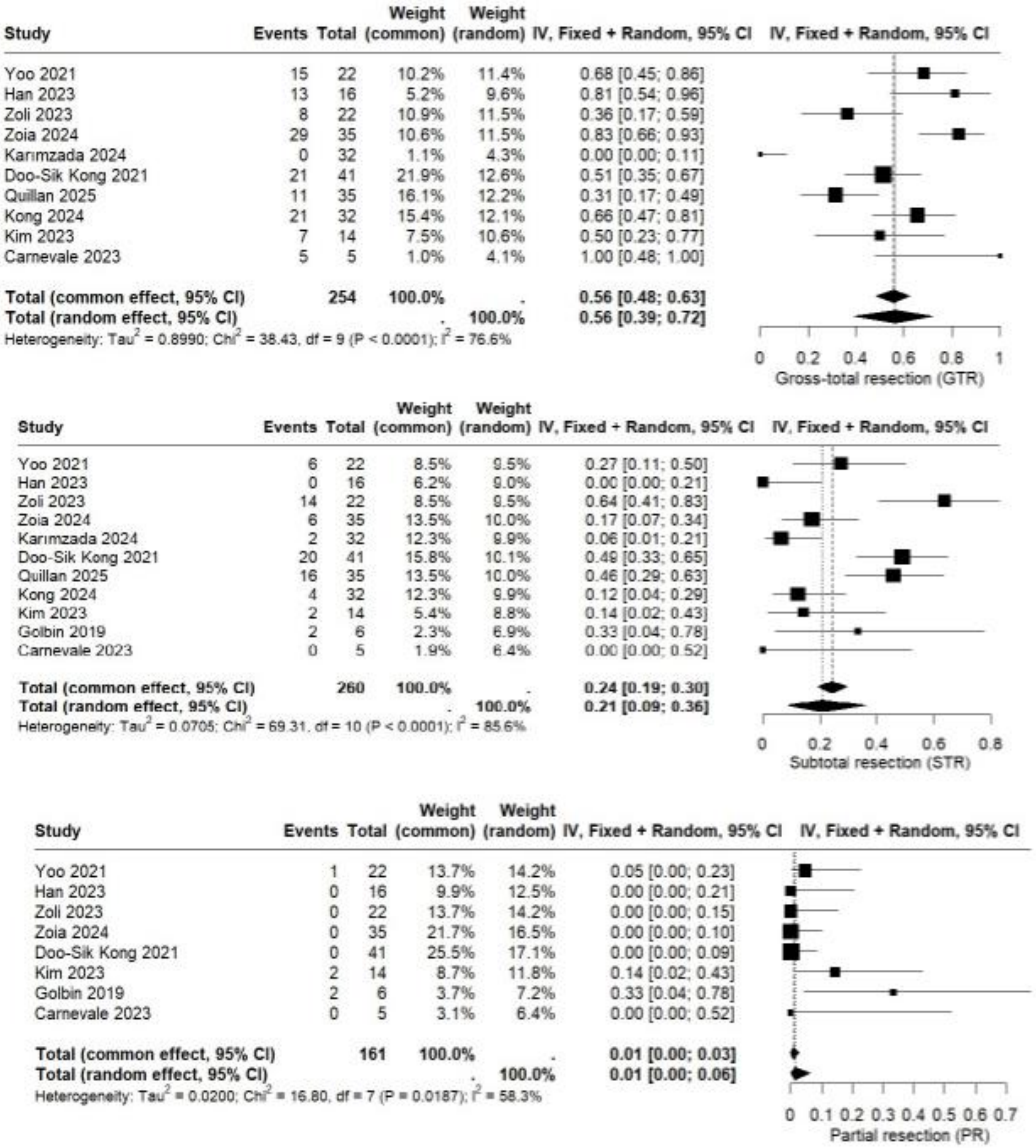

Figure S7: Forest Plot for recurrence during follow-up and mortality

| Study | Events | Total | Weight (common) | Weight (random) | IV, Fixed + Random, 95% CI |
|---|---|---|---|---|---|
| Yoo 2021 | 0 | 22 | 17.1% | 17.7% | 0.00 [0.00; 0.15] |
| Zoli 2023 | 7 | 22 | 17.1% | 17.7% | 0.32 [0.14; 0.55] |
| Zoia 2024 | 2 | 34 | 26.4% | 18.9% | 0.06 [0.01; 0.20] |
| Kong 2024 | 0 | 32 | 24.8% | 18.8% | 0.00 [0.00; 0.11] |
| Kim 2023 | 0 | 14 | 10.9% | 16.0% | 0.00 [0.00; 0.23] |
| Carnevale 2023 | 0 | 5 | 3.9% | 10.9% | 0.00 [0.00; 0.52] |
| **Total (common effect, 95% CI)** | | **129** | **100.0%** | . | **0.03 [0.01; 0.06]** |
| **Total (random effect, 95% CI)** | | . | | **100.0%** | **0.02 [0.00; 0.12]** |

Heterogeneity: $Tau^2 = 0.0499$; $Chi^2 = 25.42$, df = 5 (P = 0.0001); $I^2 = 80.3\%$

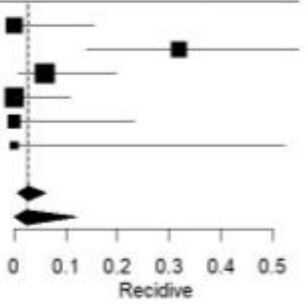


| Study | Events | Total | Weight (common) | Weight (random) | IV, Fixed + Random, 95% CI |
|---|---|---|---|---|---|
| Zoli 2023 | 2 | 22 | 15.1% | 16.6% | 0.09 [0.01; 0.29] |
| Karımzada 2024 | 0 | 32 | 21.9% | 21.1% | 0.00 [0.00; 0.11] |
| Doo-Sik Kong 2021 | 0 | 41 | 28.1% | 24.3% | 0.00 [0.00; 0.09] |
| Kong 2024 | 0 | 32 | 21.9% | 21.1% | 0.00 [0.00; 0.11] |
| Kim 2023 | 0 | 14 | 9.6% | 11.9% | 0.00 [0.00; 0.23] |
| Carnevale 2023 | 0 | 5 | 3.4% | 5.0% | 0.00 [0.00; 0.52] |
| **Total (common effect, 95% CI)** | | **146** | **100.0%** | . | **0.00 [0.00; 0.02]** |
| **Total (random effect, 95% CI)** | | . | | **100.0%** | **0.00 [0.00; 0.02]** |

Heterogeneity: $Tau^2 = 0.0052$; $Chi^2 = 7.01$, df = 5 (P = 0.2198); $I^2 = 28.7\%$

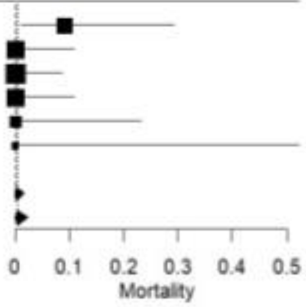

Figure S8: Baujat plot for recurrence rates during follow-up

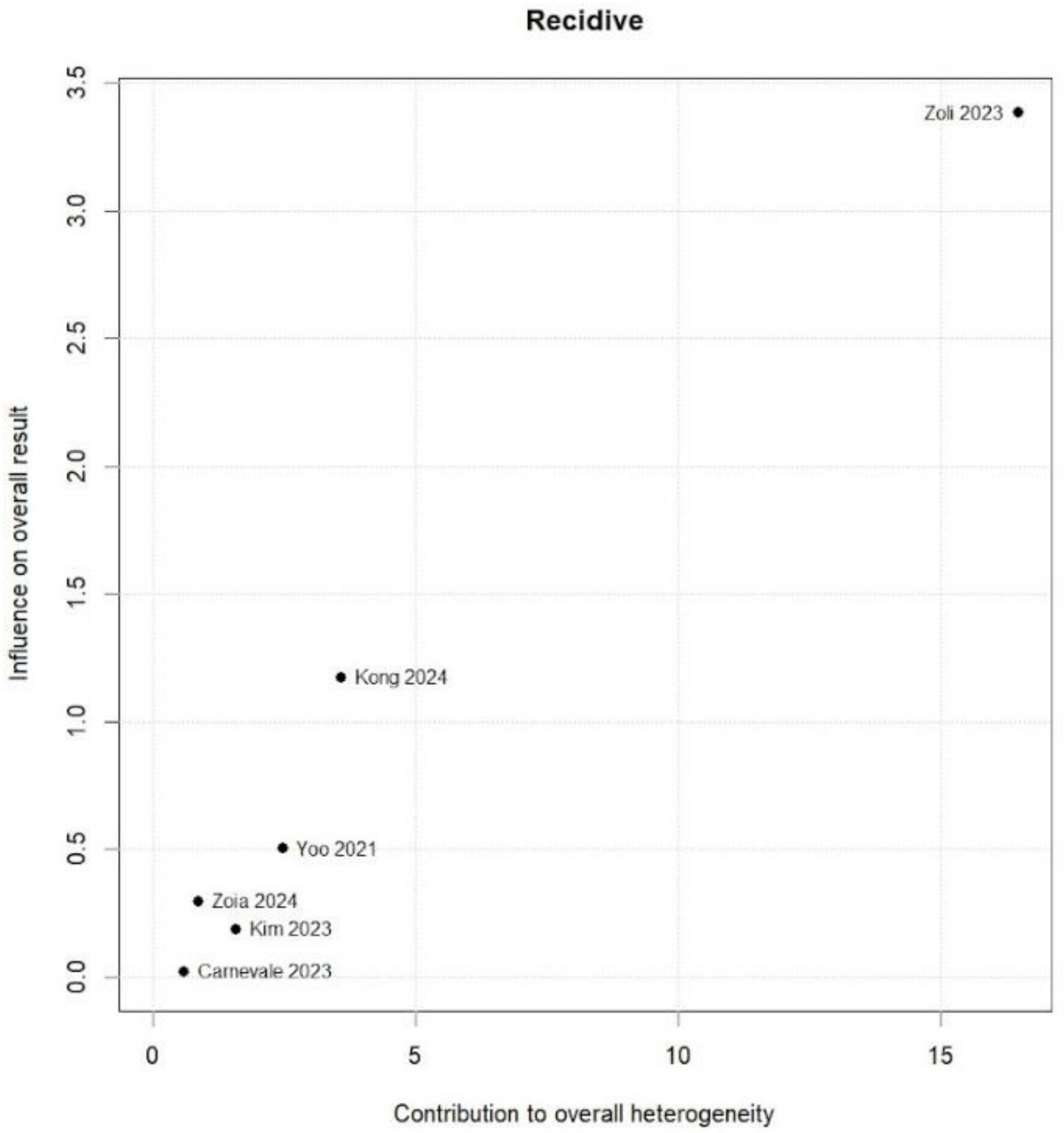

Figure S9: Leave-one-out analysis for recurrence rates during follow-up

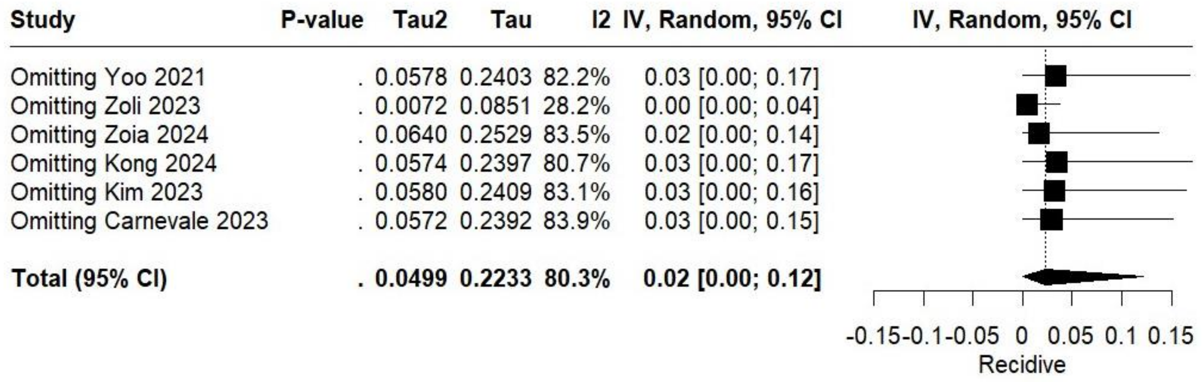


Figure S10: Forest plot for CSF leak in the treatment of meningiomas

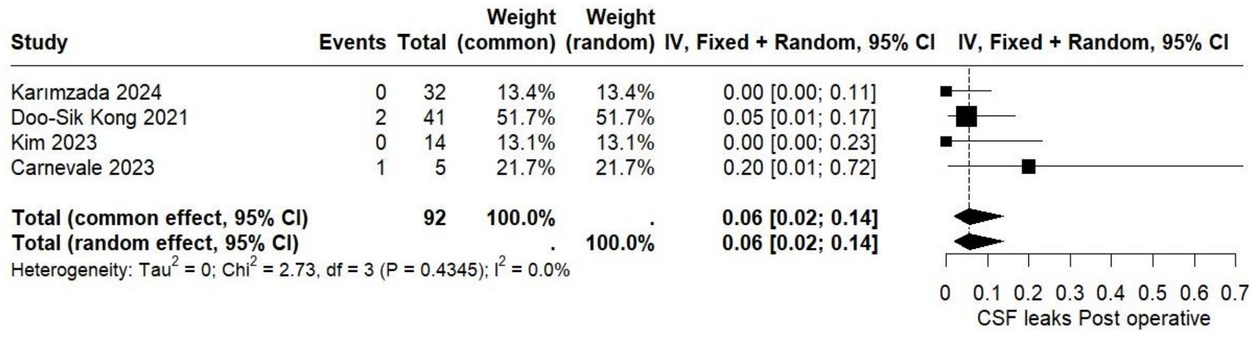


Figure S11:Forest plot for improvement in visual function in the treatment of meningiomas

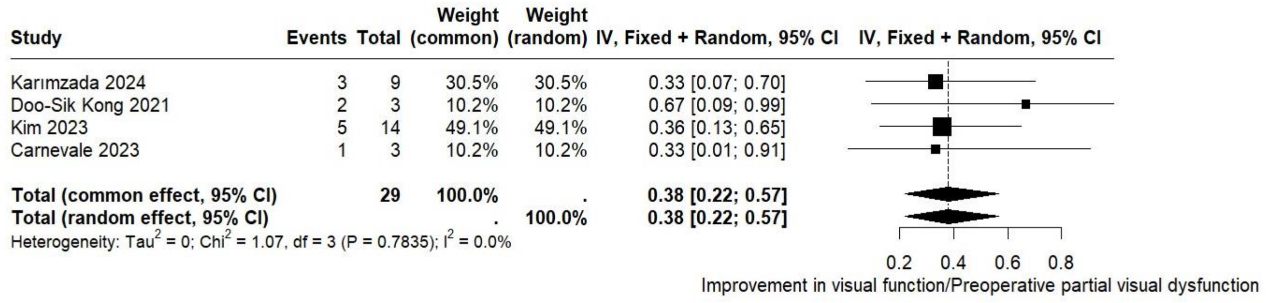

## Figure S12: Forest plot for visual dysfunction in the treatment of meningiomas

| Study | Events | Total | Weight (common) | Weight (random) | IV, Fixed + Random, 95% CI |
|---|---|---|---|---|---|
| Karımzada 2024 | 0 | 32 | 19.1% | 21.6% | 0.00 [0.00; 0.11] |
| Doo-Sik Kong 2021 | 0 | 41 | 19.1% | 21.7% | 0.00 [0.00; 0.09] |
| Kim 2023 | 1 | 14 | 36.0% | 30.8% | 0.07 [0.00; 0.34] |
| Carnevale 2023 | 1 | 3 | 25.8% | 25.9% | 0.33 [0.01; 0.91] |
| **Total (common effect, 95% CI)** | | **90** | **100.0%** | **.** | **0.06 [0.02; 0.18]** |
| **Total (random effect, 95% CI)** | | **.** | | **100.0%** | **0.06 [0.01; 0.23]** |

Heterogeneity: $Tau^2 = 1.1773$; $Chi^2 = 5.23$, df = 3 (P = 0.1559); $I^2 = 42.6\%$

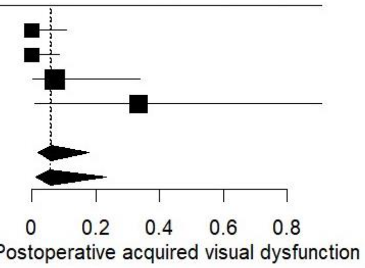


## Figure S13: Forest plot for gross-total resection (GTR) in the treatment of meningiomas

| Study | Events | Total | Weight (common) | Weight (random) | IV, Fixed + Random, 95% CI |
|---|---|---|---|---|---|
| Karımzada 2024 | 0 | 32 | 3.4% | 20.8% | 0.00 [0.00; 0.11] |
| Doo-Sik Kong 2021 | 21 | 41 | 69.7% | 30.1% | 0.51 [0.35; 0.67] |
| Kim 2023 | 7 | 14 | 23.8% | 28.8% | 0.50 [0.23; 0.77] |
| Carnevale 2023 | 5 | 5 | 3.1% | 20.3% | 1.00 [0.48; 1.00] |
| **Total (common effect, 95% CI)** | | **92** | **100.0%** | **.** | **0.49 [0.37; 0.62]** |
| **Total (random effect, 95% CI)** | | **.** | | **100.0%** | **0.41 [0.07; 0.87]** |

Heterogeneity: $Tau^2 = 4.2524$; $Chi^2 = 11.23$, df = 3 (P = 0.0106); $I^2 = 73.3\%$

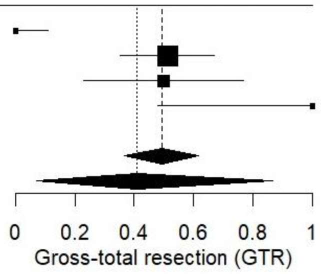


## Figure S14: Forest plot for subtotal resection (STR) in the treatment of meningiomas

| Study | Events | Total | Weight (common) | Weight (random) | IV, Fixed + Random, 95% CI |
|---|---|---|---|---|---|
| Karımzada 2024 | 2 | 32 | 13.1% | 26.3% | 0.06 [0.01; 0.21] |
| Doo-Sik Kong 2021 | 20 | 41 | 71.7% | 34.0% | 0.49 [0.33; 0.65] |
| Kim 2023 | 2 | 14 | 12.0% | 25.6% | 0.14 [0.02; 0.43] |
| Carnevale 2023 | 0 | 5 | 3.2% | 14.1% | 0.00 [0.00; 0.52] |
| **Total (common effect, 95% CI)** | | **92** | **100.0%** | **.** | **0.34 [0.23; 0.46]** |
| **Total (random effect, 95% CI)** | | **.** | | **100.0%** | **0.18 [0.05; 0.47]** |

Heterogeneity: $Tau^2 = 1.3870$; $Chi^2 = 15.26$, df = 3 (P = 0.0016); $I^2 = 80.3\%$

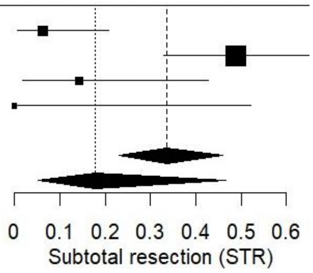

Figure S15: Forest plot for mortality in the treatment of meningiomas

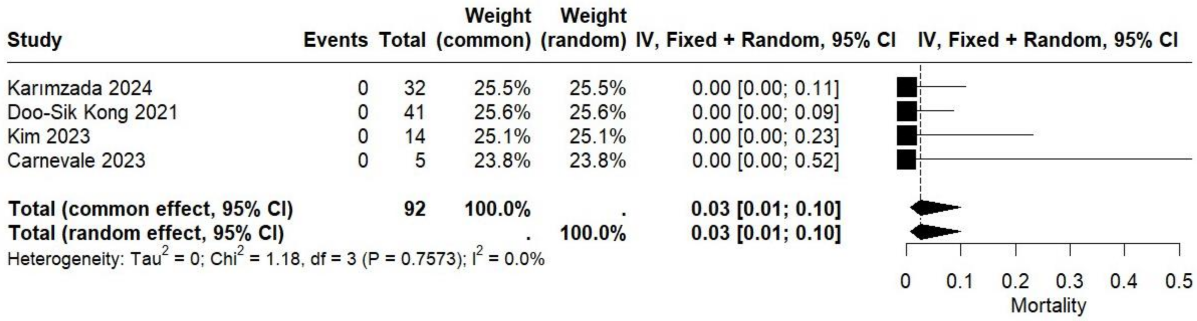


Figure S16: Funnel plot CSF leak Post Operative

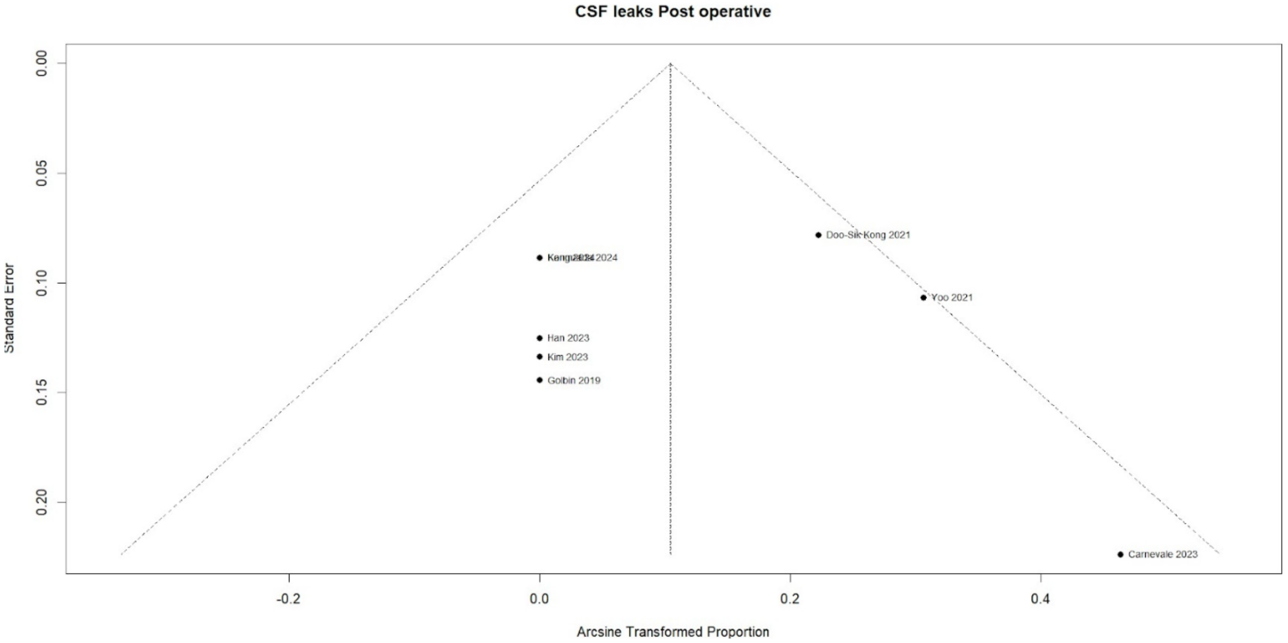

Figure S17: Funnel plot Wound infection

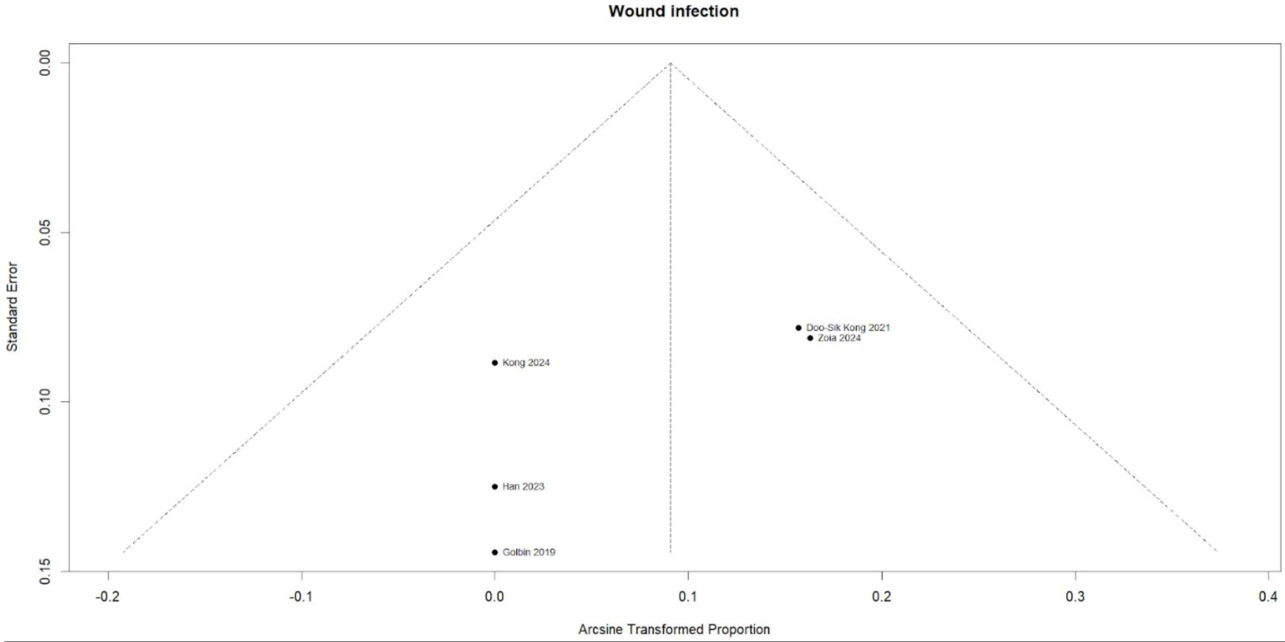


Figure S18: Funnel plot Ptosis

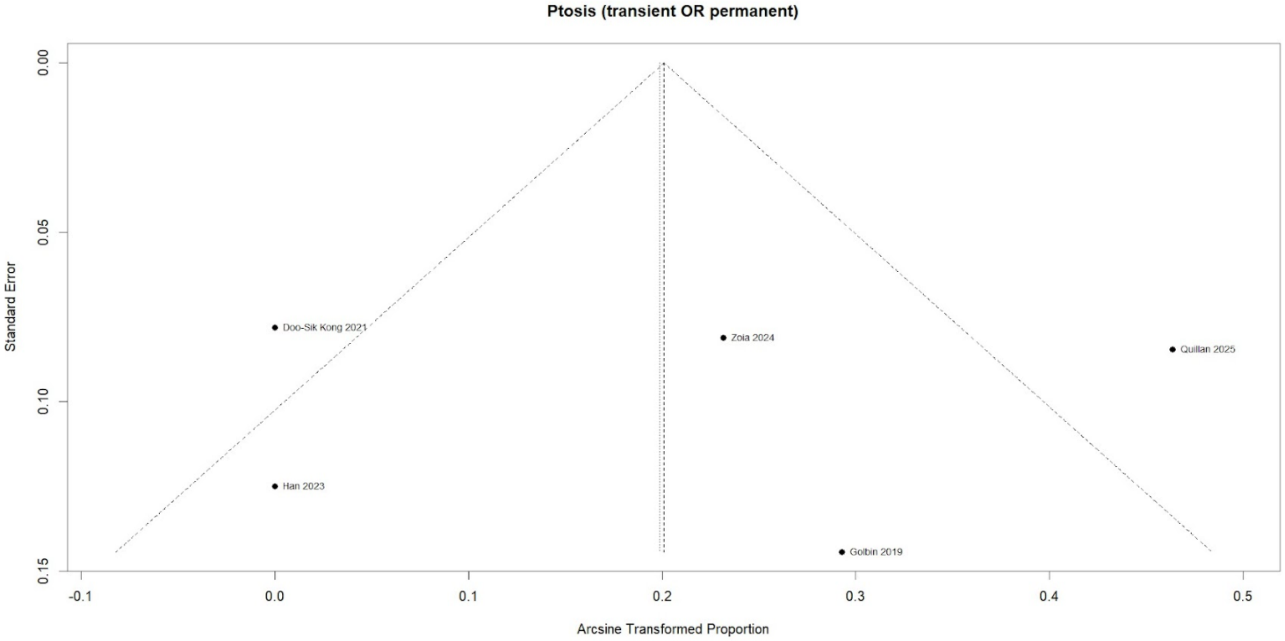

Figure S19: Funnel plot Diplopia

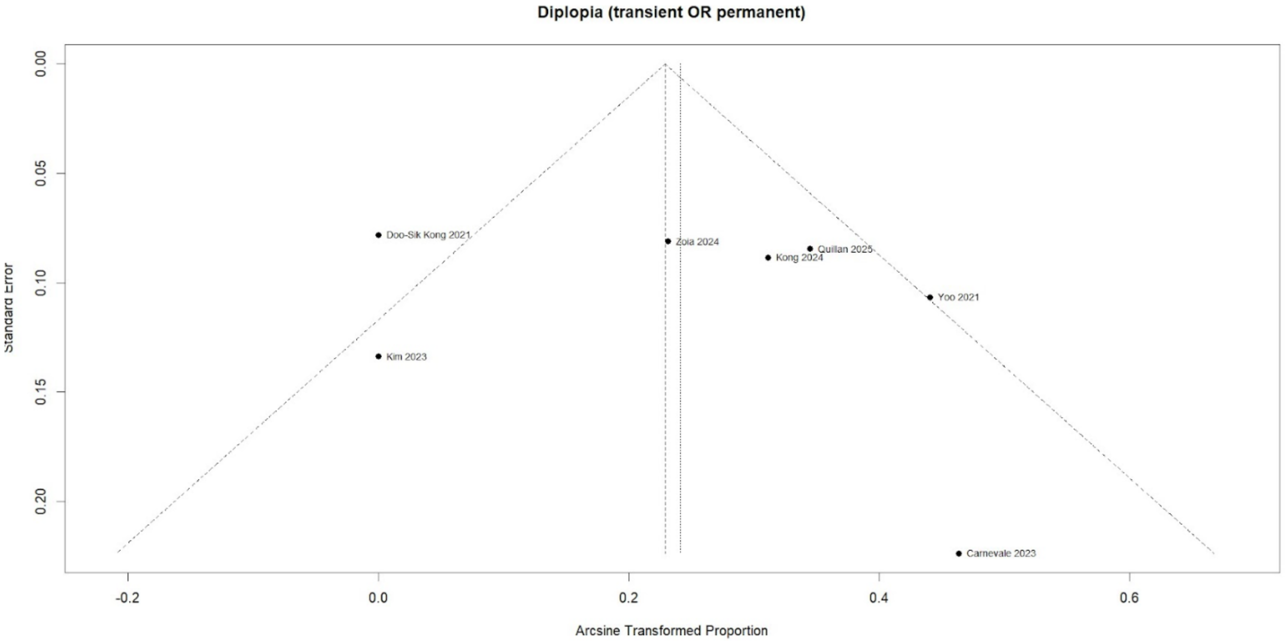


Figure S20: Funnel plot Visual improvement

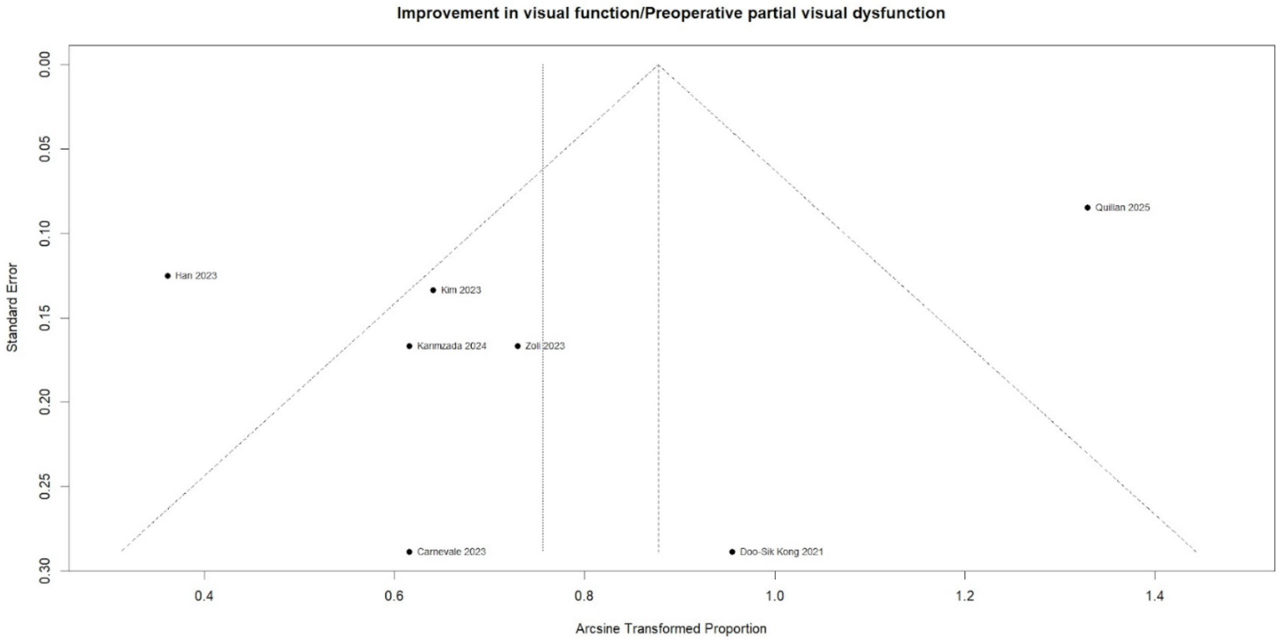

## Figure S21: Funnel plot Visual dysfunction

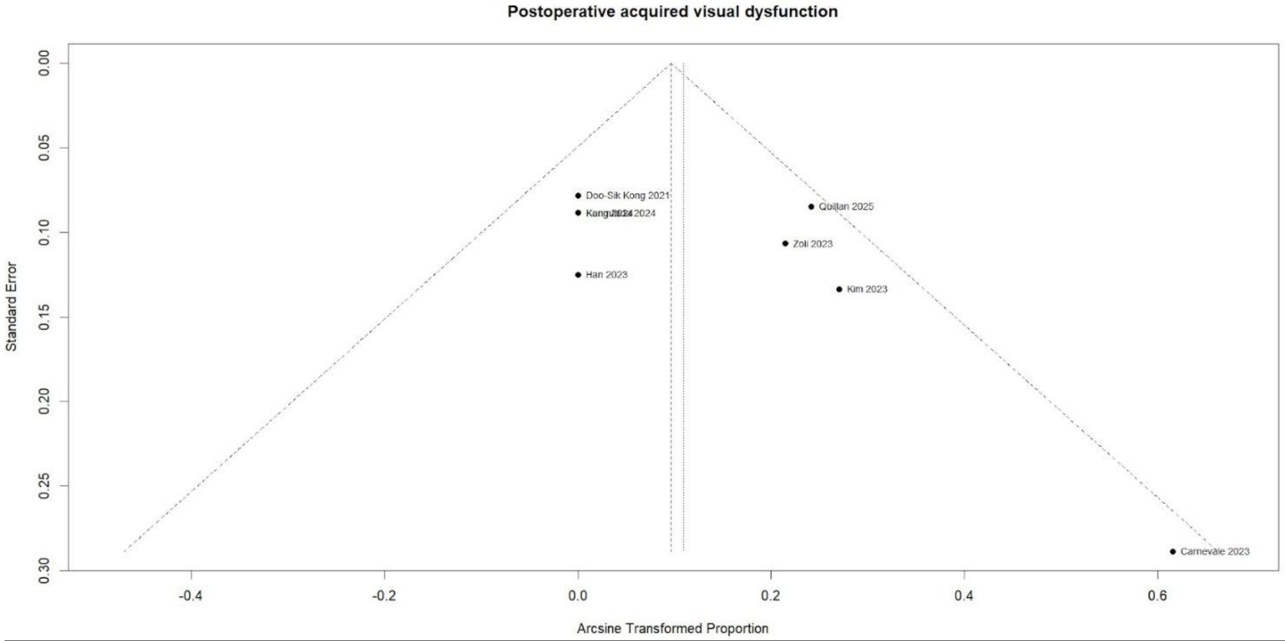


## Figure S22: Funnel plot Transient facial numbness

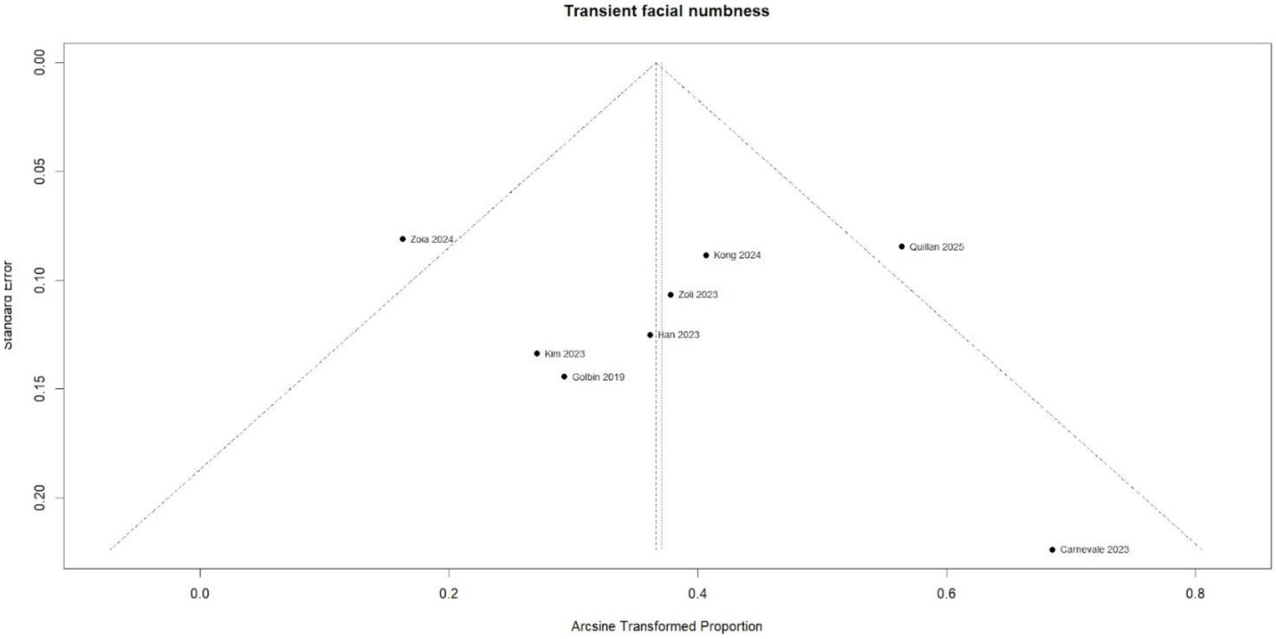

Figure S23: Funnel plot Gross-total resection (GTR)

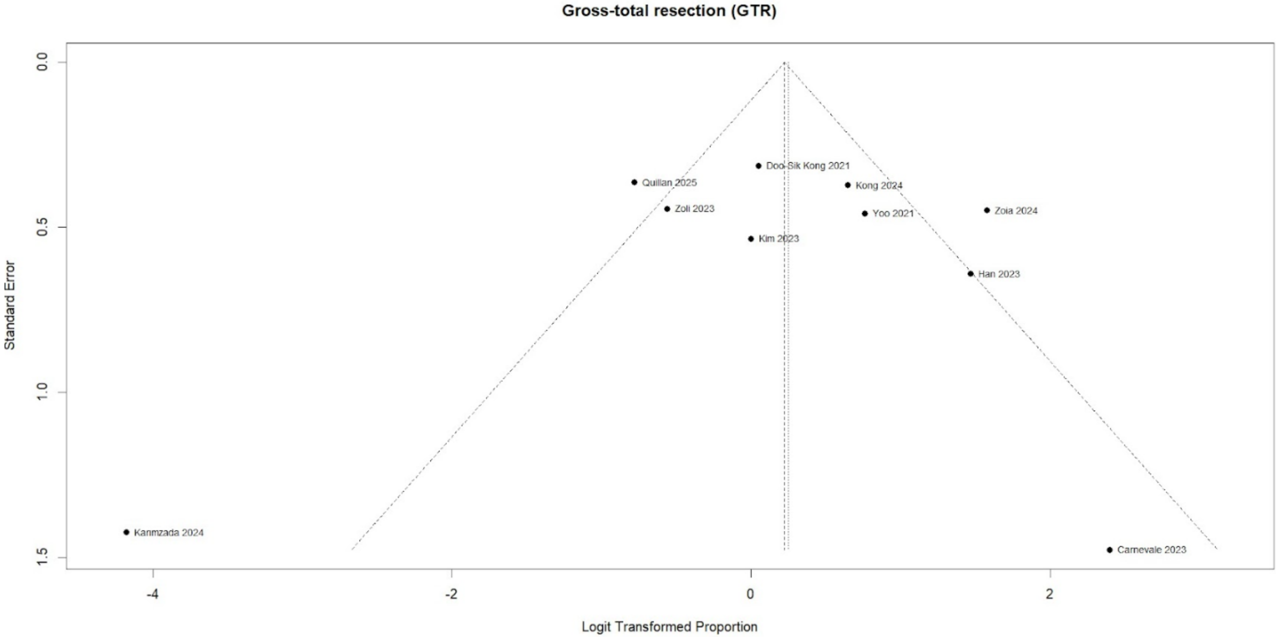


Figure S24: Funnel plot Subtotal resection (STR)

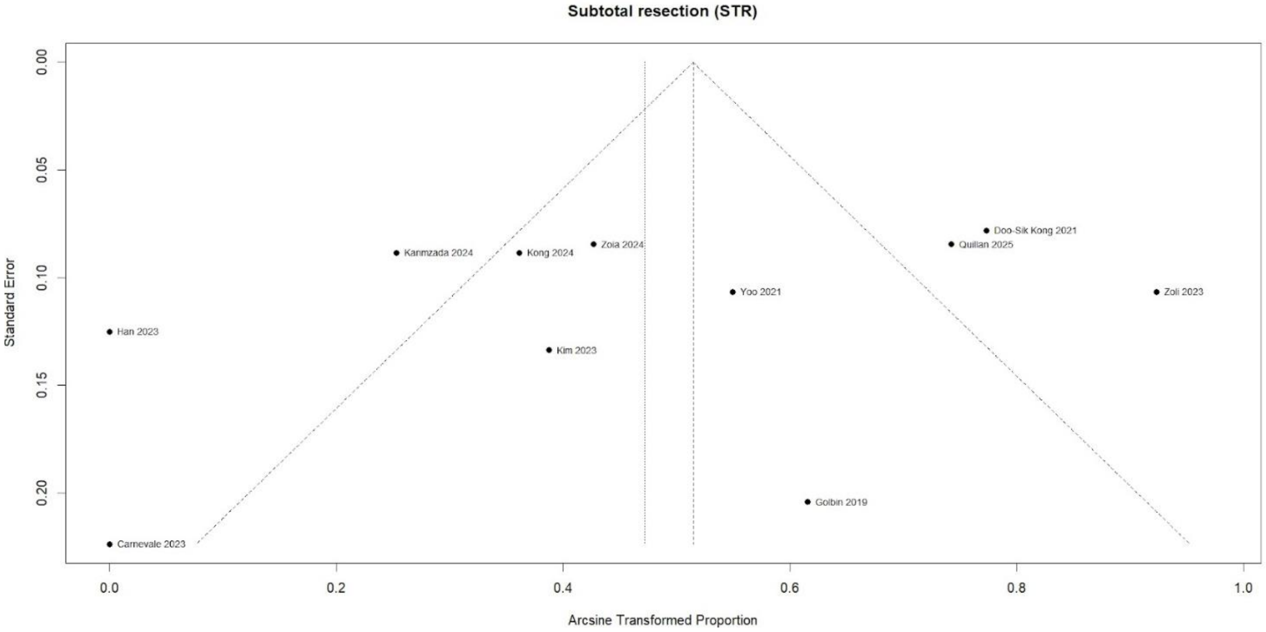

Figure S25: Funnel plot Subtotal Partial resection (PR)

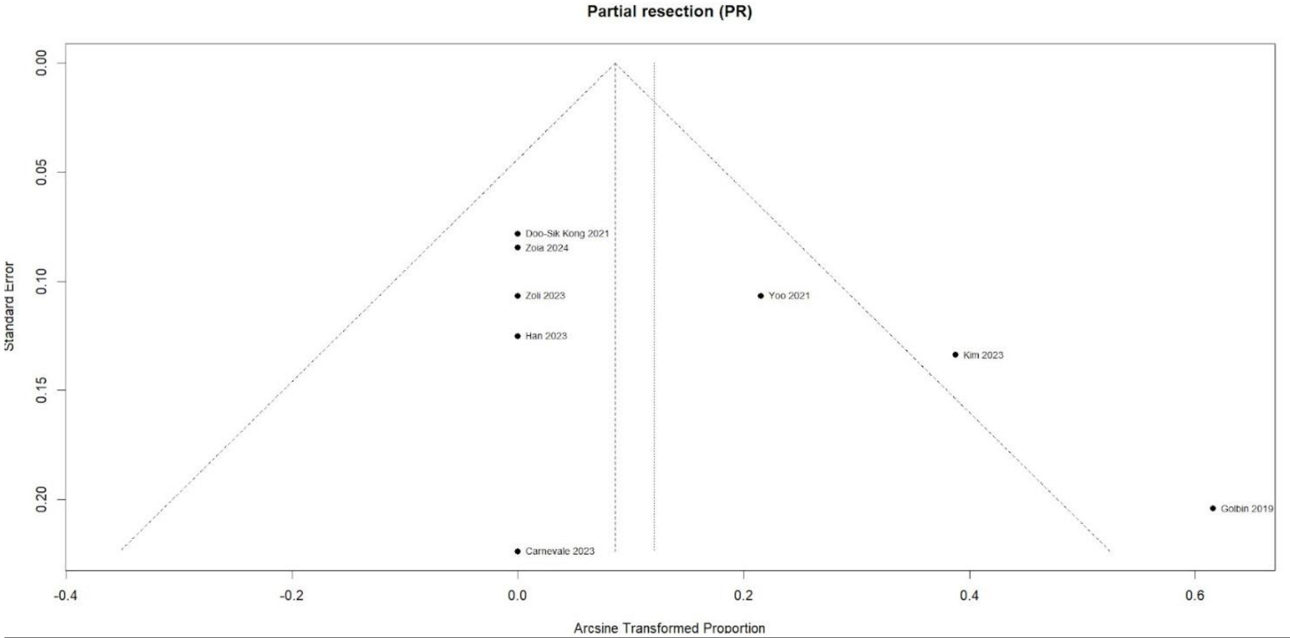


Figure S26:Funnel plot Recurrence

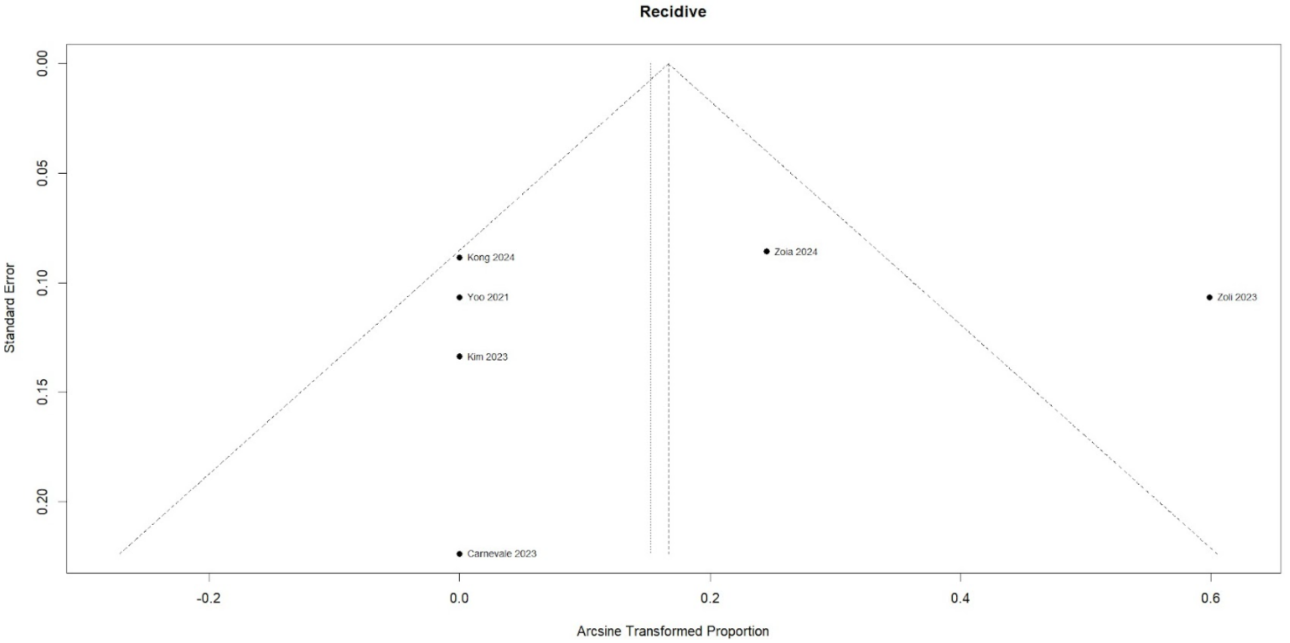

Figure S27:Funnel plot Mortality

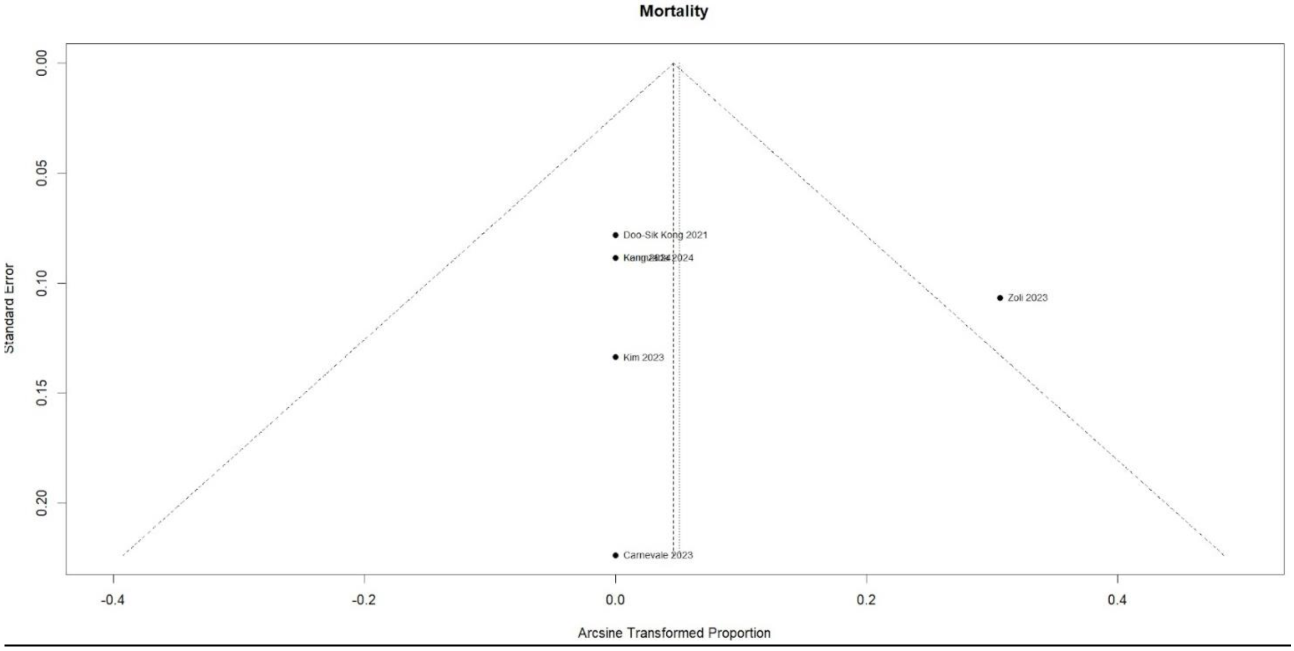


Figure S28:Funnel plot Enophthalmos

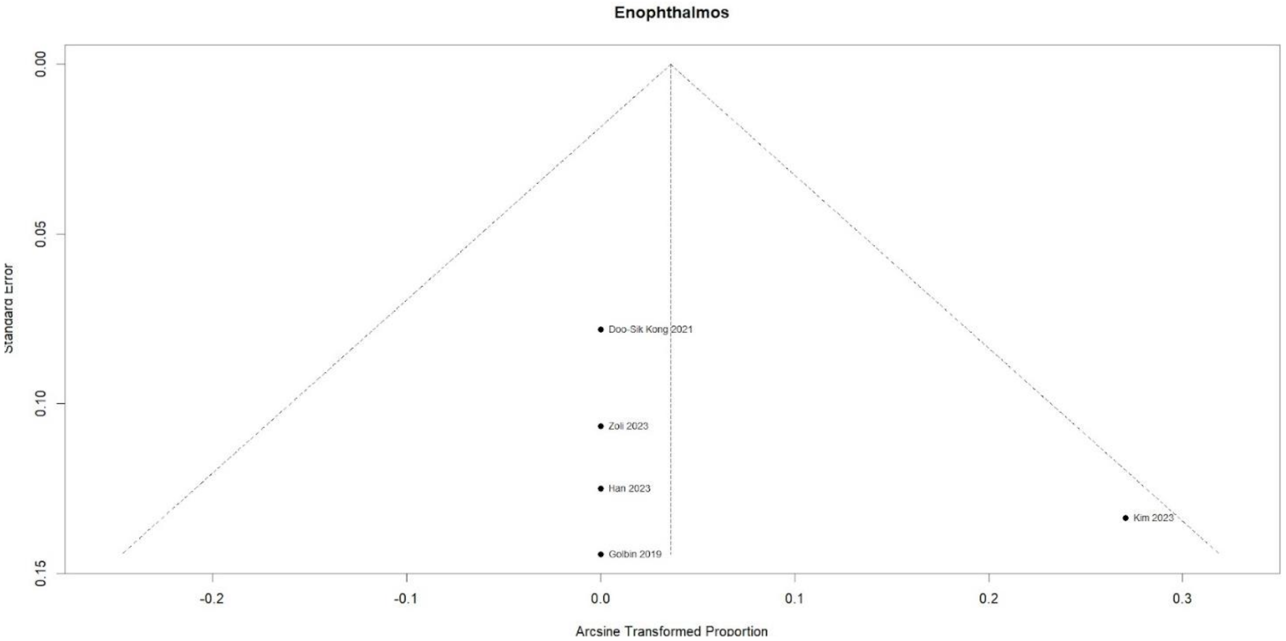

Supplement: Supplementary file 1 — Supplementary Material 1 [file 10143_2026_4321_MOESM1_ESM.pdf]
